# Supplementary material for: STANCE: a unified statistical model to detect cell-type-specific spatially variable genes in spatial transcriptomics
Source: Nat Commun. 2025 Feb 20;16:1793. doi: 10.1038/s41467-025-57117-w (PMC11842841; doi:10.1038/s41467-025-57117-w)
Supplement: Supplementary file 1 — Supplementary Information [file 41467_2025_57117_MOESM1_ESM.pdf]

Supplementary File for "STANCE: a unified statistical model to detect cell-type-specific spatially variable genes in spatial transcriptomics"

Haohao Su[1], Yuesong Wu[1], Bin Chen[2,3,4], and Yuehua Cui[1]

[1]Department of Statistics and Probability, Michigan State University, East Lansing, 48824, MI, USA

[2]Department of Pharmacology and Toxicology, Michigan State University, East Lansing, 48824, MI, USA

[3]Department of Computer Science and Engineering, Michigan State University, East Lansing, 48824, MI, USA

[4]Department of Pediatrics and Human Development, Michigan State University, Grand Rapids, 49503, MI, USA

1 Details of STANCE

1.1 The model

Our goal is to identify genes that exhibit spatial expression patterns, referred to as spatially variable genes (SVGs), specific to certain cell types. Suppose we have spatial transcriptomics expression data for q genes from n spots (or pixels) of a 2D tissue, with their spatial locations denoted as $\mathbf{s} = (s_{i1}, s_{i2})_{n\times 2}$, $i = 1, \cdots, n$. The original gene expression count data of n spots are collected and normalized through various methods to yield continuous gene expression data, denoted as $\mathbf{y} = (y_1, \cdots, y_n)^T$. Furthermore, we assume that all cells in this tissue belong to K cell types, and for each spot, the cell type compositions are estimated using existing cell type deconvolution methods, either reference-based such as RCTD [1] or [2] or reference-free such as STdeconvolve [3], denoted by $\mathbf{\Pi} = (\pi_{i1}, \cdots, \pi_{iK})_{n\times K}$, $i = 1, \cdots, n$.

With this information, we establish a variance component model[4] to elucidate the relationship between gene expressions and spatial locations, i.e.,

$$\mathbf{y}(\mathbf{s}) = \mathbf{X}(\mathbf{s})\boldsymbol{\beta} + \boldsymbol{\gamma}(\mathbf{s}) + \boldsymbol{\varepsilon}(\mathbf{s}), \tag{1}$$

where $\mathbf{X}(\mathbf{s})$ is a $n \times p$-dimensional design matrix for covariates and $\boldsymbol{\beta} = (\beta_1, \cdots, \beta_p)^T$ being a p-dimensional vector of associated coefficients (in the default case, $\mathbf{X}(\mathbf{s})\boldsymbol{\beta}$ contains only the intercept); $\boldsymbol{\gamma}(\mathbf{s})$ is a random spatial effect component, and $\boldsymbol{\varepsilon}(\mathbf{s}) = (\varepsilon_1, \cdots, \varepsilon_n)^T$ is a n-dimensional vector of random effects of the error term, following a multivariate normal distribution $MVN(\mathbf{0}, \sigma_\varepsilon^2\mathbf{I}_n)$. Model(1) is the SVG detection model commonly assumed in the literature, e.g., in SpatialDE,

SPARK or nnSVG (ref). In order to model the cell-type-specific effect, we further decompose the random effect term $\boldsymbol{\gamma}$ into K components, i.e.,

$$\boldsymbol{\gamma}(\mathbf{s}) = \boldsymbol{\pi}_1 \odot \boldsymbol{\gamma}_1(\mathbf{s}) + \cdots + \boldsymbol{\pi}_K \odot \boldsymbol{\gamma}_K(\mathbf{s}), \tag{2}$$

where $\boldsymbol{\gamma}_k(\mathbf{s})$ is an n-dimensional vector of spatial random effects contributed by cell type k, $k = 1, \cdots, K$; $\boldsymbol{\pi}_k = (\pi_{1k}, \cdots, \pi_{nk})^T$ is the k-th column of $\boldsymbol{\Pi}$, whose (i, k)-th element π_{ik} is the proportion of a specific cell type k in spot i, $i = 1, \cdots, n$; $\odot$ is the Hamadard element-wise product. We assume that cell-type spatial random effects are independent of each other and each cell-type spatial random effect follows a multivariate normal distribution,

$$\boldsymbol{\gamma}_k(\mathbf{s}) = (\gamma_k(\mathbf{s}_1), \cdots, \gamma_k(\mathbf{s}_n))^T \sim MVN(\mathbf{0}, \tau_k \mathbf{K}), k = 1, \cdots, K, \tag{3}$$

where $\mathbf{K}$ is an $n \times n$-dimensional kernel matrix capturing the spatial similarity between spots; τ_k is the variance component of spatial effect corresponding to cell type k. Combining model(1) and (2), we have

$$\mathbf{y}(\mathbf{s}) = \mathbf{X}(\mathbf{s})\boldsymbol{\beta} + \boldsymbol{\pi}_1 \odot \boldsymbol{\gamma}_1(\mathbf{s}) + \cdots + \boldsymbol{\pi}_K \odot \boldsymbol{\gamma}_K(\mathbf{s}) + \boldsymbol{\varepsilon}(\mathbf{s}). \tag{4}$$

which is the final STANCE model for cell-type-specific SVG detection. The covariance of $\mathbf{y}$ is given by

$$\mathbf{V} = \mathrm{Cov}(\mathbf{y}) = \sum_{k=1}^{K} \tau_k \boldsymbol{\Pi}_k \mathbf{K} \boldsymbol{\Pi}_k^T + \sigma_\varepsilon^2 \mathbf{I}_n = \sum_{k=1}^{K} \tau_k \boldsymbol{\Sigma}_k + \sigma_\varepsilon^2 \mathbf{I}_n, \tag{5}$$

where $\boldsymbol{\Pi}_k = \mathrm{diag}\{\boldsymbol{\pi}_k\}$ and $\boldsymbol{\Sigma}_k = \boldsymbol{\Pi}_k \mathbf{K} \boldsymbol{\Pi}_k^T$ for $k = 1, \cdots, K$.

1.2 The estimation

Denote $\boldsymbol{\tau} = (\tau_1, \cdots, \tau_K)^T$, $\boldsymbol{\phi} = (\boldsymbol{\tau}^T, \sigma_\varepsilon^2)^T$, and $\boldsymbol{\theta} = (\boldsymbol{\beta}^T, \boldsymbol{\phi}^T)^T$, then the likelihood function for $\boldsymbol{\theta}$ is

$$L(\boldsymbol{\theta}; \mathbf{y}) = (2\pi)^{-\frac{n}{2}} |\mathbf{V}|^{-\frac{1}{2}} \exp\{-\frac{1}{2}(\mathbf{y} - \mathbf{X}\boldsymbol{\beta})^T \mathbf{V}^{-1} (\mathbf{y} - \mathbf{X}\boldsymbol{\beta})\}. \tag{6}$$

Then, the log-likelihood function for $\boldsymbol{\theta}$ is given by

$$l(\boldsymbol{\theta}; \mathbf{y}) = -\frac{n}{2}\log(2\pi) - \frac{1}{2}\log|\mathbf{V}| - \frac{1}{2}(\mathbf{y} - \mathbf{X}\boldsymbol{\beta})^T \mathbf{V}^{-1} (\mathbf{y} - \mathbf{X}\boldsymbol{\beta}) \tag{7}$$

Take the first derivative of the log likelihood function with respect to $\boldsymbol{\beta}$ and set it to 0, we obtain the restricted maximum likelihood (REML) estimator as[5, 6]

$$\hat{\boldsymbol{\beta}} = (\mathbf{X}^T \mathbf{V}^{-1} \mathbf{X})^{-1} \mathbf{X}^T \mathbf{V}^{-1} \mathbf{y} \tag{8}$$

Profiling out $\boldsymbol{\beta}$ and taking logarithm gives us the restricted log likelihood function for $\boldsymbol{\phi}$,

$$\begin{aligned} l^R(\boldsymbol{\phi}; \mathbf{y}) &\propto -\frac{1}{2}\log|\mathbf{V}| - \frac{1}{2}\log|\mathbf{X}^T \mathbf{V}^{-1} \mathbf{X}| - \frac{1}{2}(\mathbf{y} - \mathbf{X}\hat{\boldsymbol{\beta}})^T \mathbf{V}^{-1} (\mathbf{y} - \mathbf{X}\hat{\boldsymbol{\beta}}) \\ &\propto -\frac{1}{2}\log|\mathbf{V}| - \frac{1}{2}\log|\mathbf{X}^T \mathbf{V}^{-1} \mathbf{X}| - \frac{1}{2}\mathbf{y}^T \mathbf{P} \mathbf{y}, \end{aligned} \tag{9}$$

where $\mathbf{P} = \mathbf{V}^{-1} - \mathbf{V}^{-1}\mathbf{X}(\mathbf{X}^T \mathbf{V}^{-1} \mathbf{X})^{-1} \mathbf{X}^T \mathbf{V}^{-1}$.

Then, the score vector of $\boldsymbol{\phi}$ is given by

$$\mathbf{S}(\boldsymbol{\phi}; \mathbf{y}) = \big(S(\tau_1; \mathbf{y}), \cdots, S(\tau_K; \mathbf{y}), S(\sigma_\varepsilon^2; \mathbf{y})\big)^T, \tag{10}$$

where

$$
\begin{aligned}
S(\tau_k; \mathbf{y}) &= \frac{\partial l^R(\boldsymbol{\phi}; \mathbf{y})}{\partial \tau_k} \\
&= -\frac{1}{2}\mathrm{tr}(\mathbf{V}^{-1}\boldsymbol{\Sigma}_k) + \frac{1}{2}\mathrm{tr}\big[(\mathbf{X}^T\mathbf{V}^{-1}\mathbf{X})^{-1}\mathbf{X}^T\mathbf{V}^{-1}\boldsymbol{\Sigma}_k\mathbf{V}^{-1}\mathbf{X}\big] + \frac{1}{2}\mathbf{y}'\mathbf{P}\boldsymbol{\Sigma}_k\mathbf{P}\mathbf{y} \\
&= -\frac{1}{2}\mathrm{tr}\Big\{\big[\mathbf{V}^{-1} - \mathbf{V}^{-1}\mathbf{X}(\mathbf{X}^T\mathbf{V}^{-1}\mathbf{X})^{-1}\mathbf{X}^T\mathbf{V}^{-1}\big]\boldsymbol{\Sigma}_k\Big\} + \frac{1}{2}\mathbf{y}'\mathbf{P}\boldsymbol{\Sigma}_k\mathbf{P}\mathbf{y} \\
&= -\frac{1}{2}\mathrm{tr}(\mathbf{P}\boldsymbol{\Sigma}_k) + \frac{1}{2}\mathbf{y}'\mathbf{P}\boldsymbol{\Sigma}_k\mathbf{P}\mathbf{y}, \qquad (11) \\
S(\sigma_\varepsilon^2; \mathbf{y}) &= \frac{\partial l^R(\boldsymbol{\phi}; \mathbf{y})}{\partial \sigma_\varepsilon^2} \\
&= -\frac{1}{2}\mathrm{tr}(\mathbf{V}^{-1}\mathbf{I}_n) + \frac{1}{2}\mathrm{tr}\big[(\mathbf{X}^T\mathbf{V}^{-1}\mathbf{X})^{-1}\mathbf{X}^T\mathbf{V}^{-1}\mathbf{I}_n\mathbf{V}^{-1}\mathbf{X}\big] + \frac{1}{2}\mathbf{y}'\mathbf{P}\mathbf{I}_n\mathbf{P}\mathbf{y} \\
&= -\frac{1}{2}\mathrm{tr}\Big\{\big[\mathbf{V}^{-1} - \mathbf{V}^{-1}\mathbf{X}(\mathbf{X}^T\mathbf{V}^{-1}\mathbf{X})^{-1}\mathbf{X}^T\mathbf{V}^{-1}\big]\mathbf{I}_n\Big\} + \frac{1}{2}\mathbf{y}'\mathbf{P}\boldsymbol{\Sigma}_k\mathbf{P}\mathbf{y} \\
&= -\frac{1}{2}\mathrm{tr}(\mathbf{P}\mathbf{I}_n) + \frac{1}{2}\mathbf{y}'\mathbf{P}\mathbf{I}_n\mathbf{P}\mathbf{y}. \qquad (12)
\end{aligned}
$$

We further compute the second derivatives of the REML log-likelihood

$$
\begin{aligned}
\frac{\partial^2 l^R(\boldsymbol{\phi}; \mathbf{y})}{\partial \tau_k \partial \tau_l} &= \frac{1}{2}\mathrm{tr}(\mathbf{P}\boldsymbol{\Sigma}_l\mathbf{P}\boldsymbol{\Sigma}_k) - \frac{1}{2}\mathbf{y}'\mathbf{P}\boldsymbol{\Sigma}_l\mathbf{P}\boldsymbol{\Sigma}_k\mathbf{P}\mathbf{y} - \frac{1}{2}\mathbf{y}'\mathbf{P}\boldsymbol{\Sigma}_k\mathbf{P}\boldsymbol{\Sigma}_l\mathbf{P}\mathbf{y} \\
&= \frac{1}{2}\mathrm{tr}(\mathbf{P}\boldsymbol{\Sigma}_l\mathbf{P}\boldsymbol{\Sigma}_k) - \mathbf{y}'\mathbf{P}\boldsymbol{\Sigma}_k\mathbf{P}\boldsymbol{\Sigma}_l\mathbf{P}\mathbf{y} \\
\frac{\partial^2 l^R(\boldsymbol{\phi}; \mathbf{y})}{\partial \tau_k \partial \sigma_\varepsilon^2} &= \frac{1}{2}\mathrm{tr}(\mathbf{P}\mathbf{I}_n\mathbf{P}\boldsymbol{\Sigma}_k) - \frac{1}{2}\mathbf{y}'\mathbf{P}\mathbf{I}_n\mathbf{P}\boldsymbol{\Sigma}_k\mathbf{P}\mathbf{y} - \frac{1}{2}\mathbf{y}'\mathbf{P}\boldsymbol{\Sigma}_k\mathbf{P}\mathbf{I}_n\mathbf{P}\mathbf{y} \\
&= \frac{1}{2}\mathrm{tr}(\mathbf{P}\mathbf{I}_n\mathbf{P}\boldsymbol{\Sigma}_k) - \mathbf{y}'\mathbf{P}\boldsymbol{\Sigma}_k\mathbf{P}\mathbf{I}_n\mathbf{P}\mathbf{y} \\
\frac{\partial^2 l^R(\boldsymbol{\phi}; \mathbf{y})}{\partial \sigma_\varepsilon^2 \partial \tau_k} &= \frac{1}{2}\mathrm{tr}(\mathbf{P}\boldsymbol{\Sigma}_k\mathbf{P}\mathbf{I}_n) - \frac{1}{2}\mathbf{y}'\mathbf{P}\boldsymbol{\Sigma}_k\mathbf{P}\mathbf{I}_n\mathbf{P}\mathbf{y} - \frac{1}{2}\mathbf{y}'\mathbf{P}\mathbf{I}_n\mathbf{P}\boldsymbol{\Sigma}_k\mathbf{P}\mathbf{y} \\
&= \frac{1}{2}\mathrm{tr}(\mathbf{P}\boldsymbol{\Sigma}_k\mathbf{P}\mathbf{I}_n) - \mathbf{y}'\mathbf{P}\boldsymbol{\Sigma}_k\mathbf{P}\mathbf{I}_n\mathbf{P}\mathbf{y} \\
\frac{\partial^2 l^R(\boldsymbol{\phi}; \mathbf{y})}{\partial \sigma_\varepsilon^4} &= \frac{1}{2}\mathrm{tr}(\mathbf{P}\mathbf{I}_n\mathbf{P}\mathbf{I}_n) - \frac{1}{2}\mathbf{y}'\mathbf{P}\mathbf{I}_n\mathbf{P}\mathbf{I}_n\mathbf{P}\mathbf{y} - \frac{1}{2}\mathbf{y}'\mathbf{P}\mathbf{I}_n\mathbf{P}\mathbf{I}_n\mathbf{P}\mathbf{y} \\
&= \frac{1}{2}\mathrm{tr}(\mathbf{P}\mathbf{I}_n\mathbf{P}\mathbf{I}_n) - \mathbf{y}'\mathbf{P}\mathbf{I}_n\mathbf{P}\mathbf{I}_n\mathbf{P}\mathbf{y}
\end{aligned}
$$

Since we have

$$
\begin{aligned}
\mathbb{E}(\mathbf{y}'\mathbf{P}\boldsymbol{\Sigma}_k\mathbf{P}\boldsymbol{\Sigma}_l\mathbf{P}\mathbf{y}) &= \mathrm{tr}(\mathbf{P}\boldsymbol{\Sigma}_l\mathbf{P}\boldsymbol{\Sigma}_k) \\
\mathbb{E}(\mathbf{y}'\mathbf{P}\boldsymbol{\Sigma}_k\mathbf{P}\mathbf{I}_n\mathbf{P}\mathbf{y}) &= \mathrm{tr}(\mathbf{P}\mathbf{I}_n\mathbf{P}\boldsymbol{\Sigma}_k) \\
\mathbb{E}(\mathbf{y}'\mathbf{P}\mathbf{I}_n\mathbf{P}\mathbf{I}_n\mathbf{P}\mathbf{y}) &= \mathrm{tr}(\mathbf{P}\mathbf{I}_n\mathbf{P}\mathbf{I}_n)
\end{aligned}
$$

then the average information matrix[7] is given by

$$
\begin{aligned}
\mathbf{AI}(\boldsymbol{\theta};\mathbf{y}) &= \begin{bmatrix}
\frac{1}{2}\Big[\frac{\partial^2 l^R(\boldsymbol{\phi};\mathbf{y})}{\partial\tau_1^2} + \mathbb{E}\big(\frac{\partial^2 l^R(\boldsymbol{\phi};\mathbf{y})}{\partial\tau_1^2}\big)\Big] & \cdots & \frac{1}{2}\Big[\frac{\partial^2 l^R(\boldsymbol{\phi};\mathbf{y})}{\partial\tau_1\partial\tau_K} + \mathbb{E}\big(\frac{\partial^2 l^R(\boldsymbol{\phi};\mathbf{y})}{\partial\tau_1\partial\tau_K}\big)\Big] & \frac{1}{2}\Big[\frac{\partial^2 l^R(\boldsymbol{\phi};\mathbf{y})}{\partial\tau_1\partial\sigma_\varepsilon^2} + \mathbb{E}\big(\frac{\partial^2 l^R(\boldsymbol{\phi};\mathbf{y})}{\partial\tau_1\partial\sigma_\varepsilon^2}\big)\Big] \\
\vdots & \ddots & \vdots & \vdots \\
\frac{1}{2}\Big[\frac{\partial^2 l^R(\boldsymbol{\phi};\mathbf{y})}{\partial\tau_K\partial\tau_1} + \mathbb{E}\big(\frac{\partial^2 l^R(\boldsymbol{\phi};\mathbf{y})}{\partial\tau_K\partial\tau_1}\big)\Big] & \cdots & \frac{1}{2}\Big[\frac{\partial^2 l^R(\boldsymbol{\phi};\mathbf{y})}{\partial\tau_K^2} + \mathbb{E}\big(\frac{\partial^2 l^R(\boldsymbol{\phi};\mathbf{y})}{\partial\tau_K^2}\big)\Big] & \frac{1}{2}\Big[\frac{\partial^2 l^R(\boldsymbol{\phi};\mathbf{y})}{\partial\tau_K\partial\sigma_\varepsilon^2} + \mathbb{E}\big(\frac{\partial^2 l^R(\boldsymbol{\phi};\mathbf{y})}{\partial\tau_K\partial\sigma_\varepsilon^2}\big)\Big] \\
\frac{1}{2}\Big[\frac{\partial^2 l^R(\boldsymbol{\phi};\mathbf{y})}{\partial\sigma_\varepsilon^2\partial\tau_1} + \mathbb{E}\big(\frac{\partial^2 l^R(\boldsymbol{\phi};\mathbf{y})}{\partial\sigma_\varepsilon^2\partial\tau_1}\big)\Big] & \cdots & \frac{1}{2}\Big[\frac{\partial^2 l^R(\boldsymbol{\phi};\mathbf{y})}{\partial\sigma_\varepsilon^2\partial\tau_K} + \mathbb{E}\big(\frac{\partial^2 l^R(\boldsymbol{\phi};\mathbf{y})}{\partial\sigma_\varepsilon^2\partial\tau_K}\big)\Big] & \frac{1}{2}\Big[\frac{\partial^2 l^R(\boldsymbol{\phi};\mathbf{y})}{\partial\sigma_\varepsilon^4} + \mathbb{E}\big(\frac{\partial^2 l^R(\boldsymbol{\phi};\mathbf{y})}{\partial\sigma_\varepsilon^4}\big)\Big]
\end{bmatrix} \\
&= \frac{1}{2}\begin{bmatrix}
\mathbf{y}'\mathbf{P}\boldsymbol{\Sigma}_1\mathbf{P}\boldsymbol{\Sigma}_1\mathbf{P}\mathbf{y} & \cdots & \mathbf{y}'\mathbf{P}\boldsymbol{\Sigma}_1\mathbf{P}\boldsymbol{\Sigma}_K\mathbf{P}\mathbf{y} & \mathbf{y}'\mathbf{P}\boldsymbol{\Sigma}_1\mathbf{P}\mathbf{I}_n\mathbf{P}\mathbf{y} \\
\vdots & \ddots & \vdots & \vdots \\
\mathbf{y}'\mathbf{P}\boldsymbol{\Sigma}_K\mathbf{P}\boldsymbol{\Sigma}_1\mathbf{P}\mathbf{y} & \cdots & \mathbf{y}'\mathbf{P}\boldsymbol{\Sigma}_K\mathbf{P}\boldsymbol{\Sigma}_K\mathbf{P}\mathbf{y} & \mathbf{y}'\mathbf{P}\boldsymbol{\Sigma}_K\mathbf{P}\mathbf{I}_n\mathbf{P}\mathbf{y} \\
\mathbf{y}'\mathbf{P}\mathbf{I}_n\mathbf{P}\boldsymbol{\Sigma}_1\mathbf{P}\mathbf{y} & \cdots & \mathbf{y}'\mathbf{P}\mathbf{I}_n\mathbf{P}\boldsymbol{\Sigma}_K\mathbf{P}\mathbf{y} & \mathbf{y}'\mathbf{P}\mathbf{I}_n\mathbf{P}\mathbf{I}_n\mathbf{P}\mathbf{y}
\end{bmatrix}.
\end{aligned} \tag{13}
$$

We can apply the Newton's method for the estimates of variance components $\boldsymbol{\phi}$ by the following iterative update[7]

$$\boldsymbol{\phi}^{(t+1)} = \boldsymbol{\phi}^{(t)} + \mathbf{AI}(\boldsymbol{\theta};\mathbf{y})\mathbf{S}(\boldsymbol{\phi}^{(t)};\mathbf{y}), \tag{14}$$

and further obtain the estimates of $\boldsymbol{\beta}$. In practice, we implement the "lmm_aireml" function in the R package 'gaston'[8] to refine the estimates more efficiently.

1.3 Hypothesis testing

1.3.1 Overall score test for detecting utSVGs

To detect utSVGs, we consider the following null and alternative hypotheses

$$\begin{cases} H_0^{(1)} : \tau_1 = \cdots = \tau_K = 0 \\ H_1^{(1)} : \text{at least one parameter is not zero} \end{cases} \tag{15}$$

Under the null hypothesis H_0^1, the null model is given by

$$\mathbf{y}(\mathbf{s}) = \mathbf{X}(\mathbf{s})\boldsymbol{\beta} + \boldsymbol{\varepsilon}(\mathbf{s}) \sim MVN(\mathbf{X}\boldsymbol{\beta}, \sigma_\varepsilon^2\mathbf{I}_n). \tag{16}$$

Then, by fitting the above null model, we obtain $\hat{\boldsymbol{\beta}} = (\mathbf{X}^T\mathbf{X})^{-1}\mathbf{X}^T\mathbf{y}$ and $\hat{\sigma}_\varepsilon^2 = \frac{(\mathbf{y}-\mathbf{X}\hat{\boldsymbol{\beta}})^T(\mathbf{y}-\mathbf{X}\hat{\boldsymbol{\beta}})}{n}$. Since $\mathbf{V}_0 = \sigma_\varepsilon^2\mathbf{I}_n$, then the corresponding $\mathbf{P}_0$ matrix is given by

$$\mathbf{P}_0 = \mathbf{V}_0^{-1} - \mathbf{V}_0^{-1}\mathbf{X}(\mathbf{X}^T\mathbf{V}_0^{-1}\mathbf{X})^{-1}\mathbf{X}^T\mathbf{V}_0^{-1} = \frac{1}{\sigma_\varepsilon^2}\big[\mathbf{I}_n - \mathbf{X}(\mathbf{X}^T\mathbf{X})^{-1}\mathbf{X}^T\big]. \tag{17}$$

According to formula(11), the REML score function for τ_k under the null is

$$S_0(\tau_k) = -\frac{1}{2}\mathrm{tr}(\mathbf{P}_0\boldsymbol{\Sigma}_k) + \frac{1}{2}\mathbf{y}^T\mathbf{P}_0\boldsymbol{\Sigma}_k\mathbf{P}_0\mathbf{y} \tag{18}$$

The REML version of score test statistic for $\boldsymbol{\tau} = (\tau_1, \cdots, \tau_K)^T$ under H_0^1 is

$$U^{(1)}(\boldsymbol{\tau}) = \sum_{k=1}^{K}\frac{1}{2}\mathbf{y}'\mathbf{P}_0\boldsymbol{\Sigma}_k\mathbf{P}_0\mathbf{y} = \frac{1}{2}\mathbf{y}^T\mathbf{P}_0\boldsymbol{\Sigma}\mathbf{P}_0\mathbf{y}, \tag{19}$$

where $\mathbf{\Sigma} = \sum_{k=1}^{K} \mathbf{\Sigma}_k$, which is a quadratic form of $\mathbf{y}$ following a mixture of chi-square distributions under the null[9, 10]. Specifically, plugging in $\mathbf{P}_0$ in (17) to the score statistic in (19), we get

$$U^{(1)}(\boldsymbol{\tau}) = \frac{1}{2}\mathbf{y}^T\mathbf{P}_0\mathbf{\Sigma}\mathbf{P}_0\mathbf{y} = \frac{1}{2\sigma_\varepsilon^4}\mathbf{y}^T\tilde{\mathbf{P}}_0\mathbf{\Sigma}\tilde{\mathbf{P}}_0\mathbf{y}, \tag{20}$$

where $\tilde{\mathbf{P}}_0 = \mathbf{I}_n - \mathbf{X}(\mathbf{X}^T\mathbf{X})^{-1}\mathbf{X}^T$ is an idempotent projection matrix.

To compute the corresponding p-value, the distribution of the test statistic $U^{(1)}(\boldsymbol{\tau})$ can be approximated by a scaled chi-square distribution $a\chi_g^2$ through the Satterthwaite approximation method[11]. Specifically, the mean e and variance v of $U^{(1)}(\boldsymbol{\tau})$ are given by

$$\begin{cases} e & = \mathbb{E}\big[U^{(1)}(\boldsymbol{\tau})\big] = \frac{1}{2\sigma_\varepsilon^2}\mathrm{tr}(\tilde{\mathbf{P}}_0\mathbf{\Sigma}) \\ v & = \mathrm{Var}\big[U^{(1)}(\boldsymbol{\tau})\big] = \frac{1}{2\sigma_\varepsilon^4}\mathrm{tr}\big[(\tilde{\mathbf{P}}_0\mathbf{\Sigma})(\tilde{\mathbf{P}}_0\mathbf{\Sigma})\big] \end{cases} \tag{21}$$

Then, we match them with the expected moments under the scaled chi-square distribution $a\chi_g^2$ to calculate the scale parameter a and the degree of freedom g, i.e.,

$$\begin{cases} e & = \mathbb{E}\big[U^{(1)}(\boldsymbol{\tau})\big] \equiv \mathbb{E}(a\chi_g^2) = ag \\ v & = \mathrm{Var}\big[U^{(1)}(\boldsymbol{\tau})\big] \equiv \mathrm{Var}(a\chi_g^2) = 2a^2g \end{cases} \tag{22}$$

Solve the above equations(22), we get

$$\begin{cases} a & = \frac{v}{2e} \\ g & = \frac{2e^2}{v} \end{cases} \tag{23}$$

Given that the true value of σ_ε^2 is unknown, replacing σ_ε^2 in (21) by its estimate $\hat{\sigma}_\varepsilon^2$ gives the estimates $\hat{e}$ and $\hat{v}$, and further the estimates $\hat{a}$ and $\hat{v}$ by

$$\begin{cases} \hat{a} & = \frac{\hat{v}}{2\hat{e}} \\ \hat{g} & = \frac{2\hat{e}^2}{\hat{v}} \end{cases} \tag{24}$$

To account for the fact that $\boldsymbol{\beta}$ and σ_ε^2 are estimated by their REML estimates under the null model, a bias correction is introduced for estimating a and g[9]. Suppose that $\boldsymbol{\beta}$ is known, the Fisher information matrix for $\boldsymbol{\phi} = (\tau_1^2, \cdots, \tau_K^2, \sigma_\varepsilon^2)^T$ can be partitioned as

$$\mathcal{I}(\tau_1, \cdots, \tau_K, \sigma_\varepsilon^2) = \begin{bmatrix} \mathcal{I}_{\boldsymbol{\tau},\boldsymbol{\tau}} & \mathcal{I}_{\boldsymbol{\tau},\sigma} \\ \mathcal{I}_{\boldsymbol{\tau},\sigma}^T & \mathcal{I}_{\sigma,\sigma} \end{bmatrix}, \tag{25}$$

where $\mathcal{I}_{\boldsymbol{\tau},\boldsymbol{\tau}}$ is the expected Fisher information for $\boldsymbol{\tau}$, whose (i,j)-th entry is $\frac{1}{2\sigma_\varepsilon^4}\mathrm{tr}\big[(\tilde{\mathbf{P}}_0\mathbf{\Sigma}_i)(\tilde{\mathbf{P}}_0\mathbf{\Sigma}_j)\big]$, $i,j = 1, \cdots, K$; $\mathcal{I}_{\sigma,\sigma} = \frac{1}{2\sigma_\varepsilon^4}\mathrm{tr}(\tilde{\mathbf{P}}_0\mathbf{I}_n\tilde{\mathbf{P}}_0\mathbf{I}_n) = \frac{1}{2\sigma_\varepsilon^4}\mathrm{tr}(\tilde{\mathbf{P}}_0)$ is the expected Fisher information for σ_ε^2; and

$$\mathcal{I}_{\boldsymbol{\tau},\sigma} = \frac{1}{2\sigma_\varepsilon^4}\big[\mathrm{tr}(\tilde{\mathbf{P}}_0\mathbf{I}_n\tilde{\mathbf{P}}_0\mathbf{\Sigma}_1), \cdots, \mathrm{tr}(\tilde{\mathbf{P}}_0\mathbf{I}_n\tilde{\mathbf{P}}_0\mathbf{\Sigma}_K)\big]^T = \frac{1}{2\sigma_\varepsilon^4}\big[\mathrm{tr}(\tilde{\mathbf{P}}_0\mathbf{\Sigma}_1), \cdots, \mathrm{tr}(\tilde{\mathbf{P}}_0\mathbf{\Sigma}_K)\big]^T. \tag{26}$$

Replacing σ_ε^2 by its REML estimator $\hat{\sigma}_\varepsilon^2$ for every partition of $\mathcal{I}(\tau_1, \cdots, \tau_K, \sigma_\varepsilon^2)$ leads to $I_{\boldsymbol{\tau},\boldsymbol{\tau}}$, $I_{\boldsymbol{\tau},\sigma}$ and $I_{\sigma,\sigma}$.

Notice that $v = \frac{1}{2\sigma_\varepsilon^4}\text{tr}\big[(\tilde{\mathbf{P}}_0\boldsymbol{\Sigma})(\tilde{\mathbf{P}}_0\boldsymbol{\Sigma})\big] = \mathbf{1}_n^T\mathcal{I}_{\tau,\tau}\mathbf{1}_n$, then we estimate v by replacing $\mathcal{I}_{\tau,\tau}$ with the efficient information

$$\tilde{v} = \mathbf{1}_n^T\tilde{I}_{\boldsymbol{\tau},\boldsymbol{\tau}}\mathbf{1}_n, \tag{27}$$

where $\tilde{I}_{\boldsymbol{\tau},\boldsymbol{\tau}} = I_{\boldsymbol{\tau},\boldsymbol{\tau}} - I_{\boldsymbol{\tau},\sigma}I_{\sigma,\sigma}^{-1}I_{\boldsymbol{\tau},\sigma}^T$ is the efficient information for $\boldsymbol{\tau}$.

Then, the bias-corrected estimates for a and g are given by

$$\begin{cases} \tilde{a} &= \frac{\tilde{v}}{2\hat{e}} \\ \tilde{g} &= \frac{2\hat{e}^2}{\tilde{v}} \end{cases}. \tag{28}$$

The corresponding p-value at significance level α can be computed by $\mathbb{P}(\frac{U^{(1)}(\boldsymbol{\tau})}{\tilde{a}} > \chi^2_{\tilde{g},\alpha})$.

1.3.2 Cell-type-specific individual score test to detect ctSVGs

For a specific cell type l, to detect its associated ctSVGs, we consider the following hypotheses,

$$\begin{cases} H_0^{(2)} : \tau_l = 0 \\ H_1^{(2)} : \tau_l > 0 \end{cases} \tag{29}$$

Under the null hypothesis $H_0^{(2)}$, we obtain the reduced model

$$\mathbf{y}(\mathbf{s}) = \mathbf{X}(\mathbf{s})\boldsymbol{\beta} + \sum_{k\neq l}^{n}\boldsymbol{\Pi}_k\boldsymbol{\gamma}_k(\mathbf{s}) + \boldsymbol{\varepsilon}(\mathbf{s}), \tag{30}$$

The variance of $\mathbf{y}$ under this null is given by $\mathbf{V}_{-l} = \sum_{k\neq l}^K \tau_k\boldsymbol{\Pi}_k\mathbf{K}\boldsymbol{\Pi}_k + \sigma_\varepsilon^2\mathbf{I}_n$, and the associated $\mathbf{P}$ matrix is

$$\mathbf{P}_{-l} = \mathbf{V}_{-l}^{-1} - \mathbf{V}_{-l}^{-1}\mathbf{X}(\mathbf{X}^T\mathbf{V}_{-l}^{-1}\mathbf{X})^{-1}\mathbf{X}^T\mathbf{V}_{-l}^{-1}.$$

Therefore, the REML version of the score test statistic for τ_l under $H_0^{(2)}$ is

$$U^{(2)}(\tau_l) = \frac{1}{2}\mathbf{y}^T\mathbf{P}_{-l}\boldsymbol{\Sigma}_l\mathbf{P}_{-l}\mathbf{y}, \tag{31}$$

which is also a quadratic form of $\mathbf{y}$ following a mixture of chi-square distributions under the null[9]. Similarly, we use the Satterthwaite method to approximate the distribution of $U^{(2)}(\tau_l)$ by a scaled chi-square distribution $a_l\chi^2_{g_l}$.

In this case, the mean and the variance of $U^{(2)}(\tau_l)$ are

$$\begin{cases} e_1 &= \mathbb{E}\big[U^{(2)}(\tau_l)\big] = \frac{1}{2}\text{tr}(\mathbf{P}_{-l}\boldsymbol{\Sigma}_l) \\ v_1 &= \text{Var}\big[U^{(2)}(\tau_l)\big] = \frac{1}{2}\text{tr}\big[(\mathbf{P}_{-l}\boldsymbol{\Sigma}_l)(\mathbf{P}_{-l}\boldsymbol{\Sigma}_l)\big] \end{cases} \tag{32}$$

Given that σ_ε^2 and $\boldsymbol{\tau}_{-l} = (\tau_1, \cdots, \tau_{l-1}, \tau_{l+1}, \cdots, \tau_K)^T$ are unknown, we replace them in $\mathbf{P}_{-l}$ by their REML estimates under the reduced model(30) to obtain $\hat{\mathbf{P}}_{-l}$ and further the estimates $\hat{e}_l$ and $\hat{v}_l$. To account for this fact, we will also perform bias correction for the test statistic $U^{(2)}(\tau_l)$[9].

We realign the elements in $\boldsymbol{\phi}_l$ to get $\boldsymbol{\phi}_l = (\sigma_\varepsilon^2, \tau_1, \cdots, \tau_{l-1}, \tau_{l+1}, \cdots, \tau_K, \tau_l)^T$. Suppose $\boldsymbol{\beta}$ is known, the Fisher information matrix for $\boldsymbol{\phi}_l$ can be partitioned as

$$\mathcal{I}(\boldsymbol{\phi}_l) = \begin{bmatrix} \mathcal{I}_{-l,-l} & \mathcal{I}_{-l,l} \\ \mathcal{I}_{-l,l}^T & \mathcal{I}_{l,l} \end{bmatrix}. \tag{33}$$

where

$$\mathcal{I}_{-l,-l} = \frac{1}{2}\begin{bmatrix} \mathrm{tr}(\mathbf{P}_{-l}\mathbf{P}_{-l}) & \mathrm{tr}(\mathbf{P}_{-l}\mathbf{P}_{-l}\mathbf{\Sigma}_1) & \cdots & \mathrm{tr}(\mathbf{P}_{-l}\mathbf{P}_{-l}\mathbf{\Sigma}_K) \\ \mathrm{tr}(\mathbf{P}_{-l}\mathbf{\Sigma}_1\mathbf{P}_{-l}) & \mathrm{tr}(\mathbf{P}_{-l}\mathbf{\Sigma}_1\mathbf{P}_{-l}\mathbf{\Sigma}_1) & \cdots & \mathrm{tr}(\mathbf{P}_{-l}\mathbf{\Sigma}_1\mathbf{P}_{-l}\mathbf{\Sigma}_K) \\ \vdots & \vdots & \ddots & \vdots \\ \mathrm{tr}(\mathbf{P}_{-l}\mathbf{\Sigma}_K\mathbf{P}_{-l}) & \mathrm{tr}(\mathbf{P}_{-l}\mathbf{\Sigma}_K\mathbf{P}_{-l}\mathbf{\Sigma}_1) & \cdots & \mathrm{tr}(\mathbf{P}_{-l}\mathbf{\Sigma}_K\mathbf{P}_{-l}\mathbf{\Sigma}_K), \end{bmatrix} \tag{34}$$

is the expected Fisher information for $\boldsymbol{\tau}_{-l}$;

$$\mathcal{I}_{l,l} = \frac{1}{2}\mathrm{tr}(\mathbf{P}_{-l}\mathbf{\Sigma}_l\mathbf{P}_{-l}\mathbf{\Sigma}_l) \tag{35}$$

is the expected Fisher information for σ_ε^2; and

$$\mathcal{I}_{-l,l} = \frac{1}{2}\left[\mathrm{tr}(\mathbf{P}_{-l}\mathbf{\Sigma}_l\mathbf{P}_{-l}), \mathrm{tr}(\mathbf{P}_{-l}\mathbf{\Sigma}_l\mathbf{P}_{-l}\mathbf{\Sigma}_1), \cdots, \mathrm{tr}(\mathbf{P}_{-l}\mathbf{\Sigma}_l\mathbf{P}_{-l}\mathbf{\Sigma}_K)\right]^T. \tag{36}$$

Replacing $\mathbf{P}_{-l}$ by $\hat{\mathbf{P}}_{-l}$ evaluated at $\hat{\sigma}_\varepsilon^2$, $\hat{\boldsymbol{\tau}}_{-l}$ leads to $I(\boldsymbol{\phi}_l)$ under the null model. Notice that $v_l = \mathcal{I}_{l,l}$, then we estimate v_l by the efficient information

$$\tilde{I}_{l,l} = I_{l,l} - I_{-l,l}^T I_{-l,-l}^{-1} I_{-l,l} \equiv \tilde{v}_l. \tag{37}$$

Then, the bias-corrected estimates for a_l and g_l are given by

$$\begin{cases} \tilde{a}_l & = \tilde{v}_l / 2\hat{e}_l \\ \tilde{g}_l & = 2\hat{e}_l^2 / \tilde{v}_l \end{cases}. \tag{38}$$

The corresponding p-value at significance level α can be computed by $\mathbb{P}(\frac{U^{(2)}(\tau_l)}{\tilde{a}_l} > \chi^2_{\tilde{g}_l,\alpha})$.

1.4 Kernel matrix

The kernel matrix, which represents the spatial correlation pattern of spots within the target tissue, is crucial for parameter estimation and statistical inference. In our approach, we employ a Gaussian kernel defined as $\mathbf{K}(\mathbf{s}_i, \mathbf{s}_j) = \exp\{-\frac{|\mathbf{s}_i - \mathbf{s}_j|^2}{h}\}$, where $|\mathbf{s}_i - \mathbf{s}_j|$ denotes the Euclidean distance between spots $\mathbf{s}_i$ and $\mathbf{s}_j$, and h is the bandwidth. The Euclidean distance is spatial rotation invariant, and the STANCE model links the gene expression pattern and spatial location through the Gaussian kernel. Thus, not like CTSV[12], C-SIDE[13], and spVC[14], the estimation and testing results are invariant to spatial rotation and translation.

To determine the appropriate bandwidth h, we adhere to the method proposed by spatialPCA[15]. Specifically, for datasets with 5000 spots or fewer, non-parametric Sheather-Jones' bandwidths[16] are computed for each gene, and the median of these values is used as the common bandwidth h; for datasets with more than 5000 spots, Silverman's rule of thumb[17] is modified to calculate a bandwidth for each gene, and the median of these gene-specific bandwidths is used as the common bandwidth h. One can also apply the cosine kernel with different parameters and get different p-values, then these p-values can be combined with the Cauchy combination rule as implemented in SPARK.

2 Additional details on real data analysis

2.1 Quality control and cell type deconvolution

The human HER2+ breast cancer tumor dataset[18] can be found at https://zenodo.org, and we used sample H1 as an example, which contains gene expression count data for 15,030 genes over 613 spots. Following the paper, we applied Stereoscope[19], a reference-based deconvolution method, to get the cell type composition of each spot. We used the major tier annotation with 8 cell types, including myeloid cells, T cells, B cells, epithelial cells, plasma cells, endothelial cells, cancer-associated fibroblasts (CAFs), and Perivascular like cells (PVL cells). We then removed 21 genes identified as ring-pattern technical artifacts in the original study following the work of Stereoscope[19] and low-expressed genes that do not express in more than 10% spots, resulting in 10,053 genes.

The human kidney cancer dataset can be found at https://data.mendeley.com. We focused on the tumor core sample of patient PD47171, which contains expression count data for 36,601 genes across 3,008 spots, we used CARD[2], a reference-based cell type deconvolution tool, to obtain the cell type compositions. Specifically, we followed the default quality control procedure of CARD to remove low-expressed genes and spots. We obtained the cell type proportions for 12 cell types, including B cells, plasma cells, T cells, natural killer (NK) cells, endothelial cells (EC), renal cell carcinoma (RCC) cells, non-proximal tubule epithelial (Epi_non-PT) cells, proximal tubule epithelial (Epi_PT) cells, fibrobalast cells, myeloid cells, plasmacytoid dendritic cells (pDC), as well as mast cells, across 2,917 spots. We then removed mitochondrial genes and low-expressed genes that do not express at more than 10% spots, resulting in 7,270 genes.

The mouse olfactory bulb (MOB) dataset [20] can be found at https://www.spatialresearch.org. We focused on MOB replicate #8, which consists of expression count data for 15,928 genes across 262 pixels (spots). We then deconvolved the MOB data using STdeconvolve[3], a reference-free and unsupervised cell type deconvolution tool. Before cell type deconvolution, we followed the procedure of the original work of STdeconvolve to clean the dataset, resulting in 7,365 genes and 260 spots.

2.2 Domain detection analysis

The spatial domain detection analysis for human HER2+ breast cancer tumor dataset is conducted using five domain detection methods, including sueratPCA, SpatialPCA, BayesSpace, Stlearn and SpaceFlow. For each method, the default gene expression features were replaced with utSVGs and ctSVGs by STANCE, and SVGs by SPARK-G to compare domain detection accuracy across different input features.

SeuratPCA uses gene expression as input, applies principle component analysis (PCA) to reduce the gene expression matrix to top 30 PCs, and employs the Louvain clustering algorithm to get 7 spatial domains.

SpatialPCA uses gene expression and spatial location as input, applies probabilistic PCA to reduce the gene expression matrix to top 20 spatial PCs, and employs the walktrap clustering algorithm to get 7 spatial domains.

BayesSpace uses gene expression and spatial location as input, reduces the gene expression matrix to top 30 PCs, and infers the latent cluster labels to get 7 spatial domains.

Stlearn uses gene expression, spatial location and HE image as input to develop spatial morphological gene expressions (SMEs) by averaging gene expression values by neighboring points, reduces the SMEs to top 50 PCs, and employs the Leiden clustering algorithm to get 7 spatial domains.

SpaceFlow uses gene expression and spatial location as input to construct a spatial expression graph, and then uses graph convolutional networks with a learning rate 10^{-4} to generate low-dimension embeddings that capture local expression patterns, then employs the Leiden clustering algorithm to get 7 spatial domains.

2.3 Gene set enrichment analyses

The gene set enrichment analyses were conducted using the "gProfiler2" package for the human breast cancer and kidney cancer datasets[21]. We used the 'gost' function to assess the significance of pathways associated with each group of ctSVGs, based on GO, KEGG, and Reactome gene sets. Specifically, we adapted the default "g_SCS" algorithm for multiple testing correction and set the significance level to be 0.05.

2.4 Computational time and memory usage

The STANCE first-stage overall test, which employs a score statistic on a reduced model, is designed to be computationally efficient. However, the second-stage individual tests, which analyze each cell type separately, may require additional computational resources. In addition, the computational time scales approximately cubically with the number of spots due to the use of the kernel matrix, which may impose computational challenges for any kernel-based methods such as STANCE and SPARK[22].

For the MOB[20] replicate #8 dataset comprising 7,365 genes, 260 spots, and 12 cell types (post-quality control and cell type deconvolution), the computational performance of various methods was evaluated on a desktop equipped with an Intel Core i7-13650HX CPU and 24 GB of RAM. The memory was calculated using the "bench_memory" function in the R `bench` package, which computes the cumulative memory usage. The results are summarized as follows:

- SPARK-G[22]: Completed in approximately 100 seconds (1.65 minutes), with a total memory usage of 3.63 GB.
- SPARK-X[23]: Completed in approximately 9.84 seconds, with a total memory usage of 170 MB.
- STANCE stage 1 overall test: Completed in approximately 31.58 seconds, which includes 14.27 seconds for creating the STANCE object, normalization, constructing the kernel matrix, and computing covariance matrices for all variance components. The total memory usage was 448 MB.
- STANCE stage 2 individual tests: Required approximately 985 seconds (16.4 minutes) to analyze all 828 utSVGs detected by the STANCE stage 1 test across all 12 deconvolved cell types. The total memory usage was 95.6 GB.

- spVC[14]: Required approximately 1.13 hours to complete the entire process, excluding the time needed for pre-selecting boundary points and creating triangulations based on the boundary. The total memory usage was 117 GB.
- CTSV[12]: Demonstrated intensive computational demands when analyzing all 7,365 genes. For comparison, it required approximately 5.16 minutes to process a subset of 10 genes. Taking the average time as 0.516 min/gene, it requires about 63 hours. The total memory usage for CTSV could not be measured using the "bench_memory".

Supplementary figures

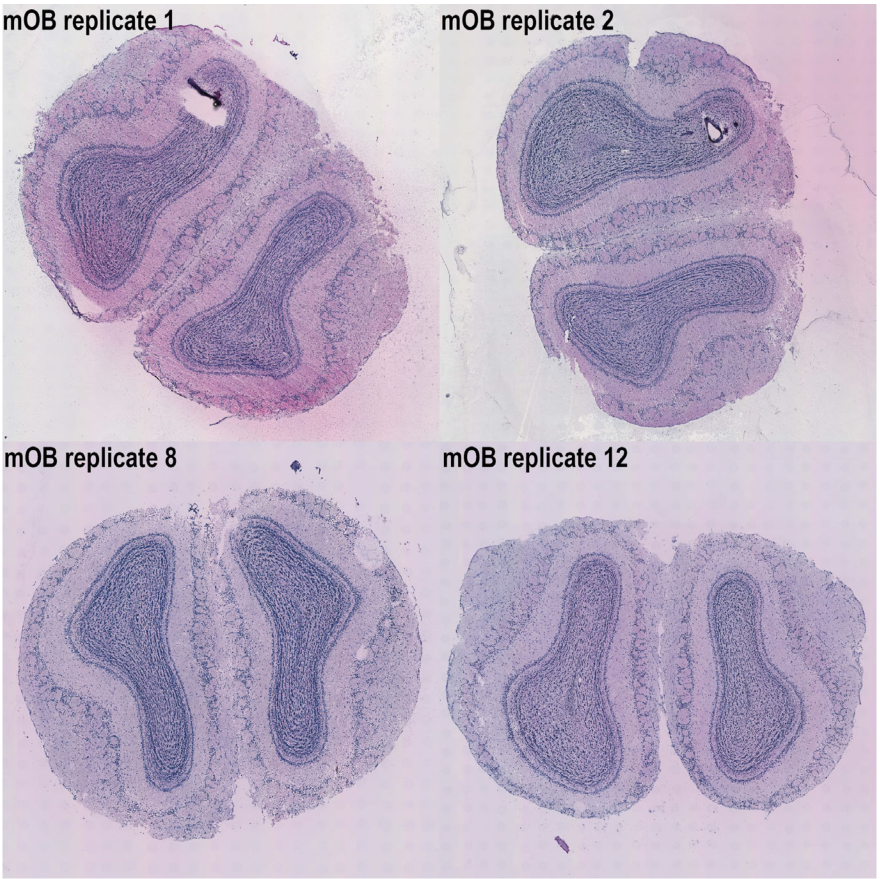

Fig. S1: **Images of four mouse olfactory bulb tissue sections.** Displayed are the Hematoxylin and eosin-stained brightfield images of mouse olfactory bulb tissue replicate sample 1, 2, 8, and 12[20] (https://www.spatialresearch.org). The variability in tissue orientation and positioning during sample preparation highlights the need for rotation-invariant statistical methods in spatial transcriptomics.

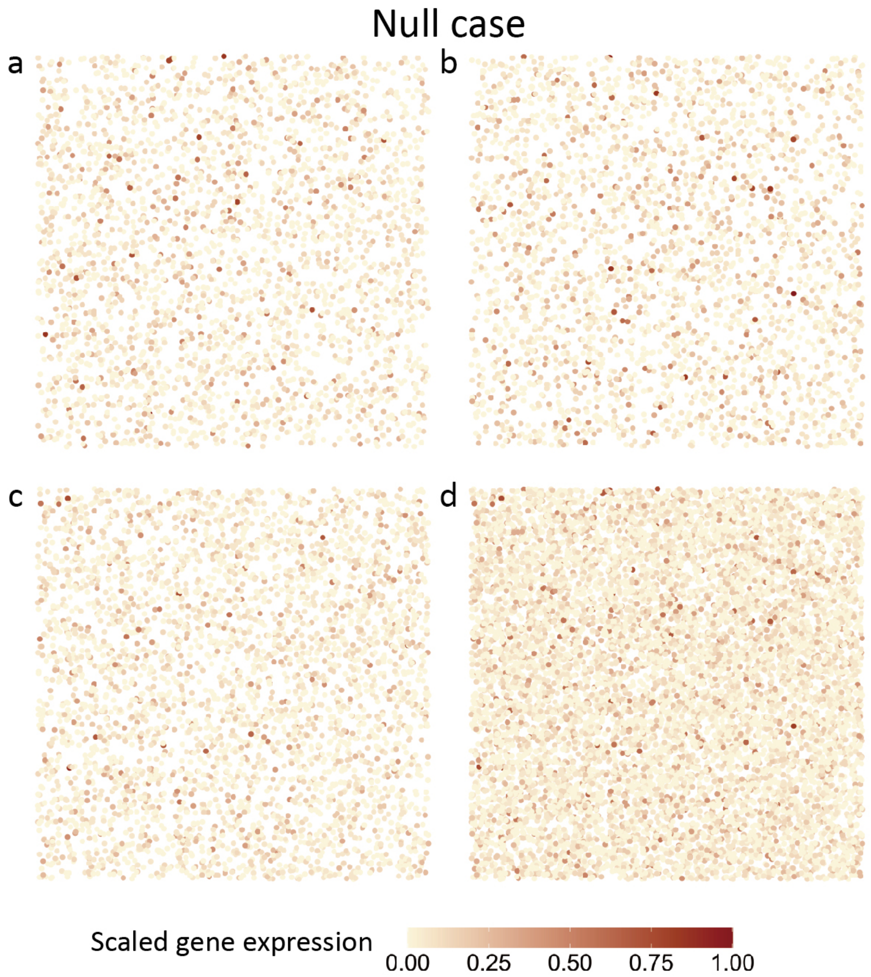

Fig. S2: **The spatial expression patterns of a representative non-spatial gene (both non-SVG and non-ctSVG).**

a. The spatial expression pattern of cell type 1 shows a random spatial pattern and hence is not a ctSVG.

b. The spatial expression pattern of cell type 2 shows a random spatial pattern and hence is not a ctSVG.

c. The spatial expression pattern of cell type 3 shows a random spatial pattern and hence is not a ctSVG.

d. The spatial expression pattern of the three combined cell types shows a random spatial pattern and hence is not an SVG.

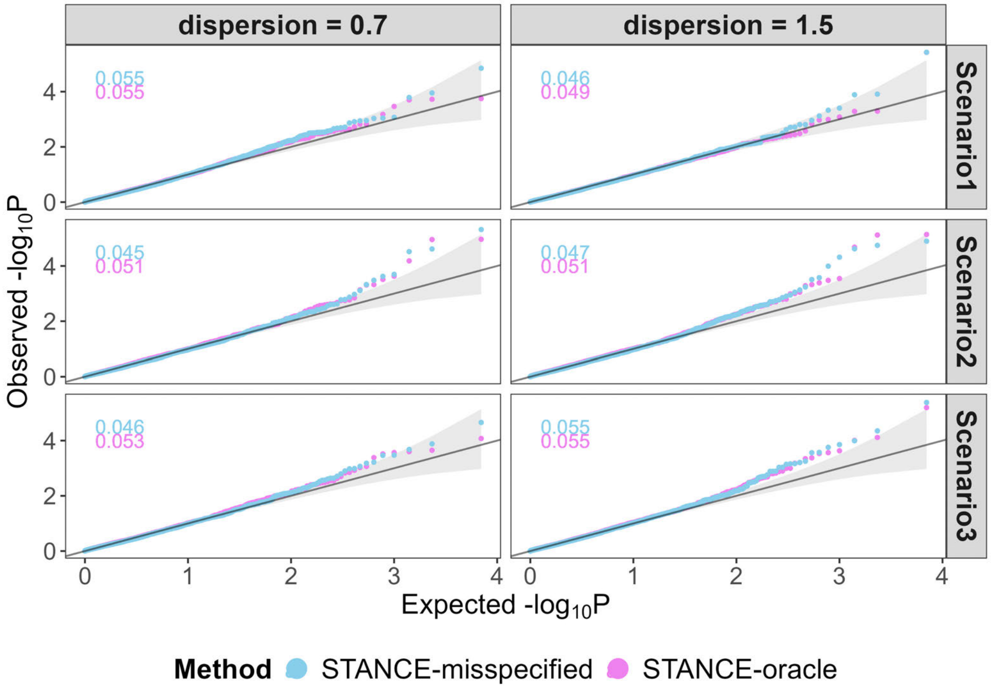

Fig. S3: **Simulation results to assess the type I error control for the overall test in Simulation 1 with mis-specified cell type compositions.** The Q-Q plots of the observed $-\log_{10} P$ against the expected $-\log_{10} P$ for different methods are displayed across various dispersion parameters and scenarios. Each sub-figure also includes the empirical type I error rate of different methods, shown in the top left corner, with colors matching those in the legend. The light gray region represents the 95% error band, illustrating the range in which 95% of points are expected to fall under the null hypothesis, assuming the p-values follow the expected uniform distribution. When the cell type compositions are miss-deconvolved, the STANCE overall test still controls the type I error well.

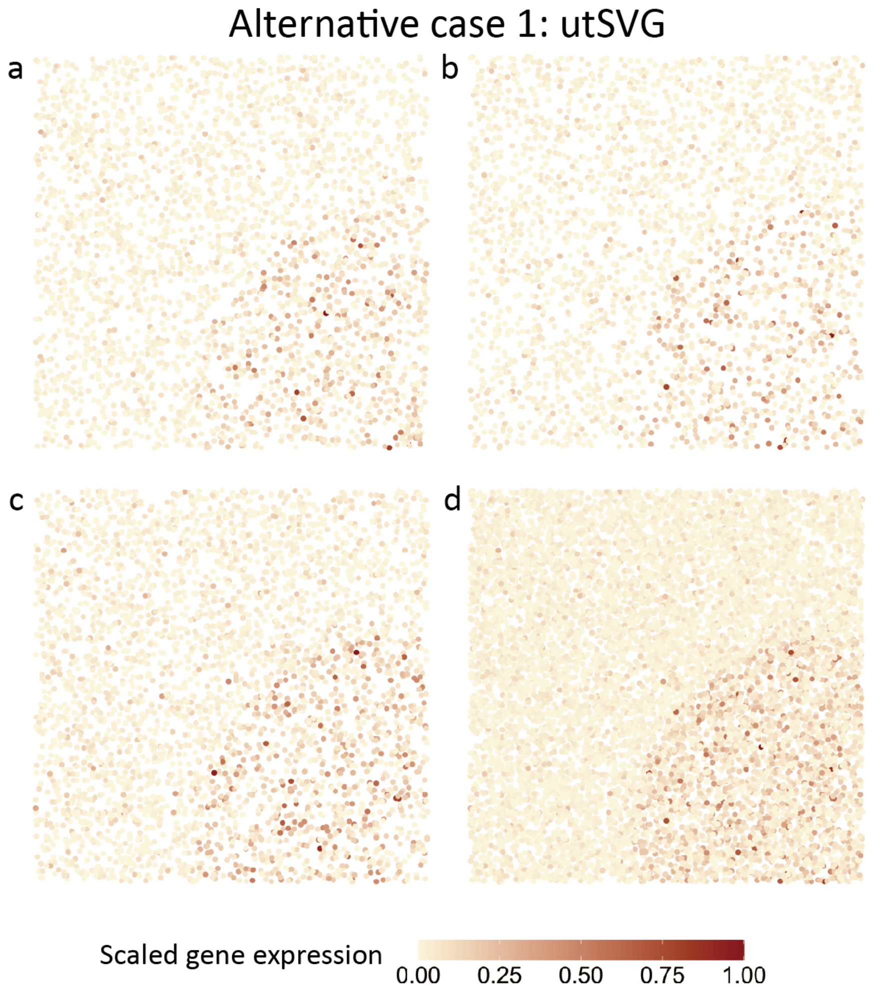

Fig. S4: **The spatial patterns of a representative utSVG's expression.** **a-d** show the expression pattern for cell type 1-3 and the combined expression pattern, respectively. The gene is a ctSVG and SVG, hence a utSVG.

a. Cell type 1-specific pattern displaying scaled gene expression for cells in cell type 1. The gene expression in domain $D2$ is higher than that in the other domains. Hence, it is a cell type 1 ctSVG.

b. Cell type 2-specific pattern displaying scaled gene expression for cells in cell type 2. The gene expression in domain $D2$ is higher than that in the other domains. Hence, it is a cell type 2 ctSVG.

c. Cell type 3-specific pattern displaying scaled gene expression for cells in cell type 3. The gene expression in domain $D2$ is higher than that in the other domains. Hence, it is a cell type 3 ctSVG.

d. Combined pattern displaying scaled gene expression combining all three cell types. The gene expression in domain $D2$ is higher than that in the other domains. Hence, it is an SVG.

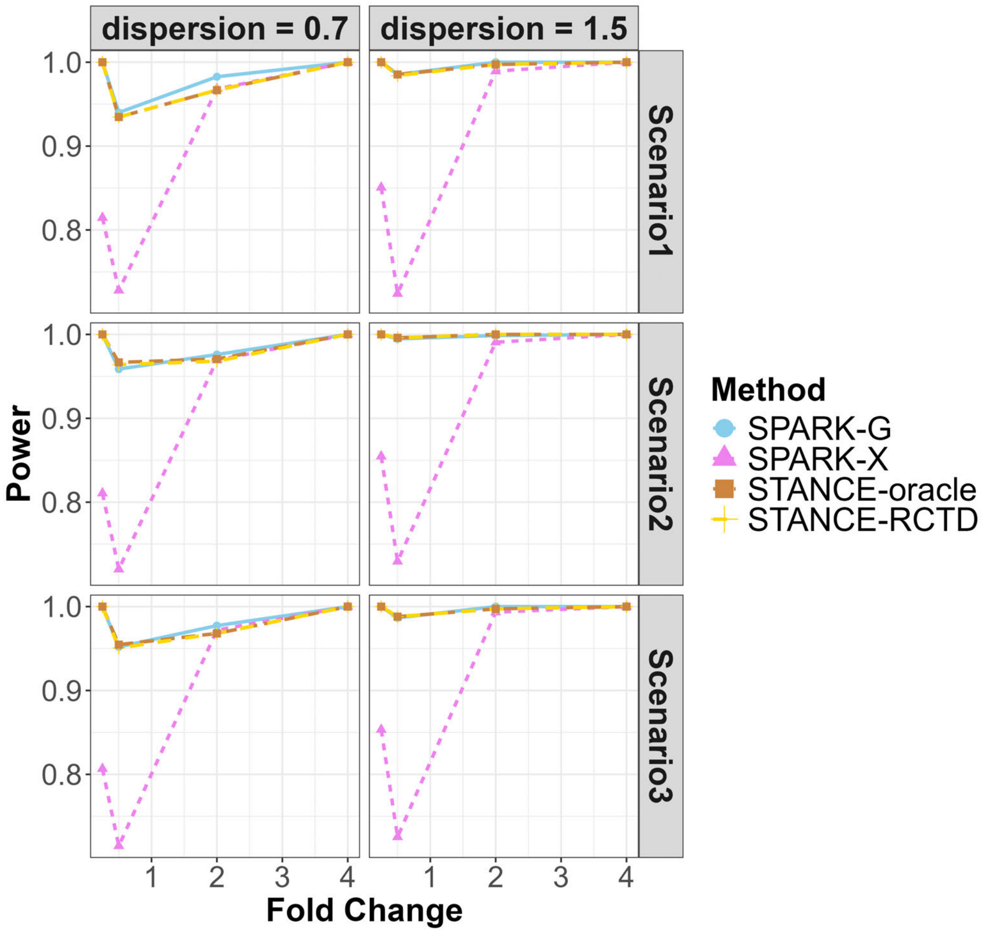

Fig. S5: **Simulation results to assess the power of the overall test under Alternative Case 1 in Simulation 1.** Simulation 1 Alternative Case 1 involves both SVGs and ctSVGs. The plots display power values against the fold changes in gene expression for different methods, across various dispersion parameters and cell type composition scenarios, under the significance level of 0.05. In this simulation, STANCE-RCTD and STANCE-oracle perform similarly to SPARK-G and better than SPARK-X. Source data are provided as a Source Data file.

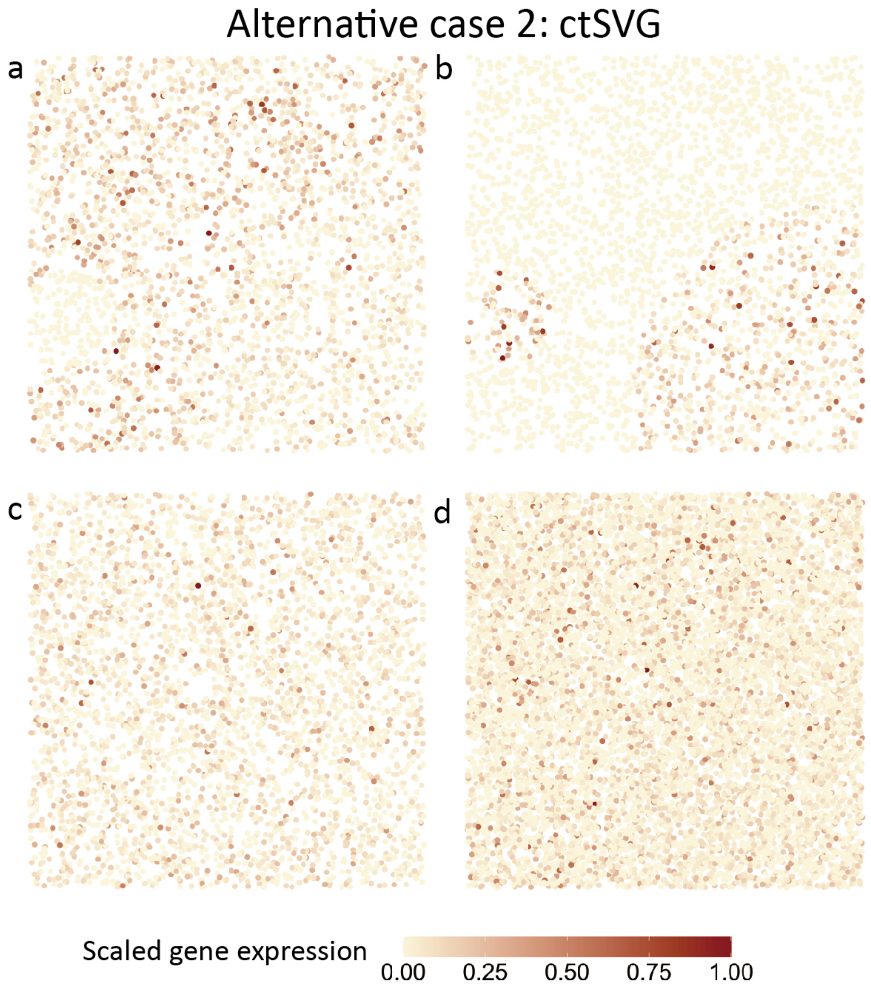

Fig. S6: **The spatial expression patterns of a representative ctSVG.** The gene is a cell type 1 and 2 ctSVG but not an SVG and cell type 3 ctSVG.

a. Cell type 1-specific pattern displaying scaled gene expression for cells in cell type 1. The gene does not express in domain $D3$. Hence, it is a cell type 1-specific ctSVG.

b. Cell type 2-specific pattern displaying scaled gene expression for cells in cell type 2. The gene does not express in domain $D1$. Hence, it is a cell type 2-specific ctSVG.

c. Cell type 3-specific pattern displaying scaled gene expression for cells in cell type 3. This representative gene displays random spatial pattern from the perspective of cell type 3. Hence, it is not a cell type 3-specific ctSVG.

d. Combined pattern displaying scaled gene expression for all cells. This representative gene displays random spatial pattern from the combined perspective. Hence, it is not an SVG.

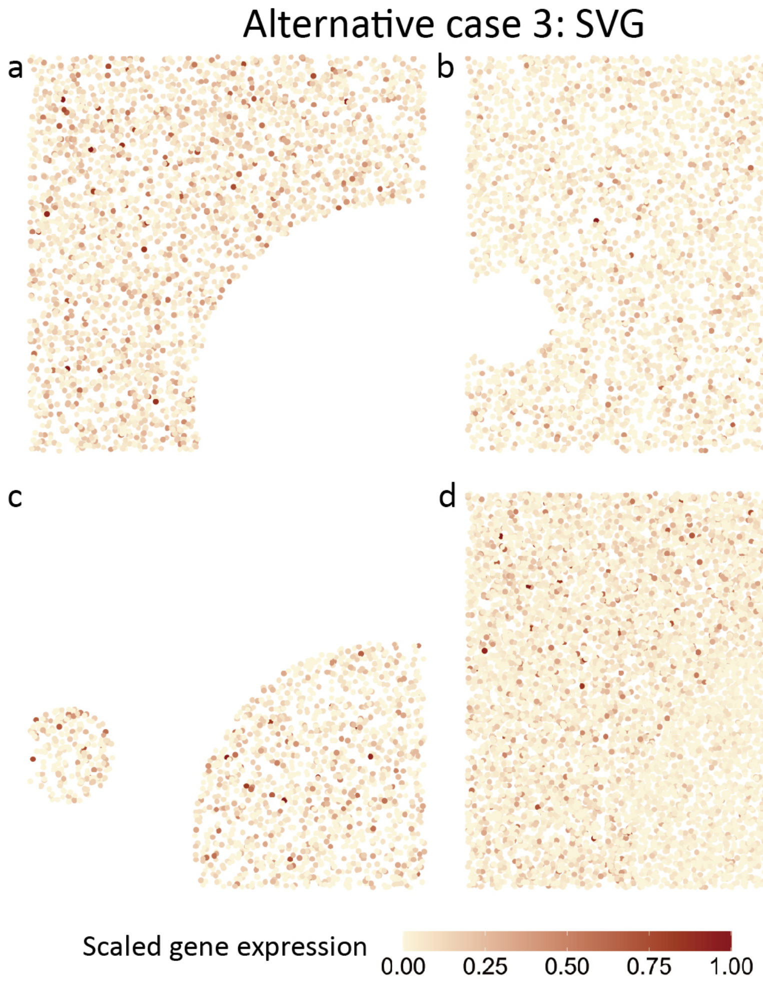

Fig. S7: **The spatial expression patterns of a representative SVG.**

a. Cell type 1-specific pattern displaying scaled gene expression for cells in cell type 1. This gene displays random spatial pattern in domain D1 and D3, but no expression in D2. Hence, it is not a cell type 1-specific ctSVG.

b. Cell type 2-specific pattern displaying scaled gene expression for cells in cell type 2. This gene displays random spatial pattern in domain D1 and D2, but no expression in D3. Hence, it is not a cell type 2-specific ctSVG.

c. Cell type 3-specific pattern displaying scaled gene expression for cells in cell type 3. This gene displays random spatial pattern in domain D2 and D3 but not in D1. Hence, it is not a cell type 3-specific ctSVG.

d. Combined pattern displaying scaled gene expression in all cells. The gene expression in domain $D2$ is lower than that in the other domains. Hence, it is an SVG.

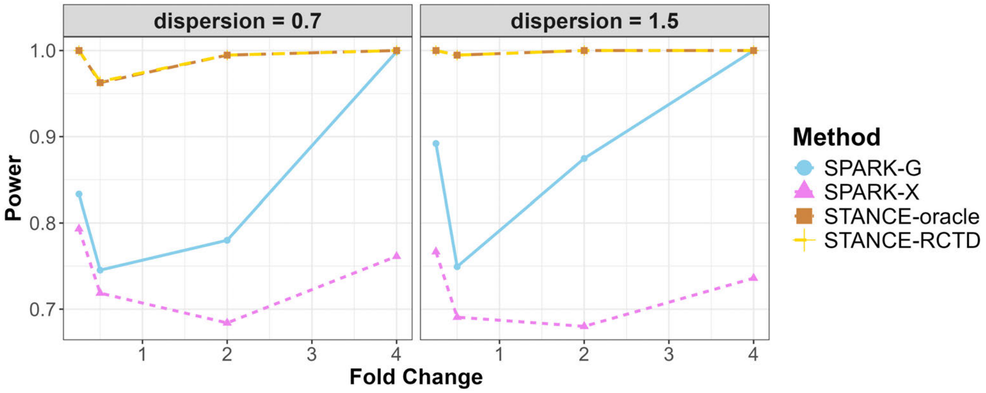

Fig. S8: **Simulation results to assess the power of the overall test under Alternative Case 3 in Simulation 1.** Simulation 1 alternative Case 3 involves only SVGs. The plots display power values against the fold changes in gene expression for different methods, across various dispersion parameters, under the significance level of 0.05. Source data are provided as a Source Data file.

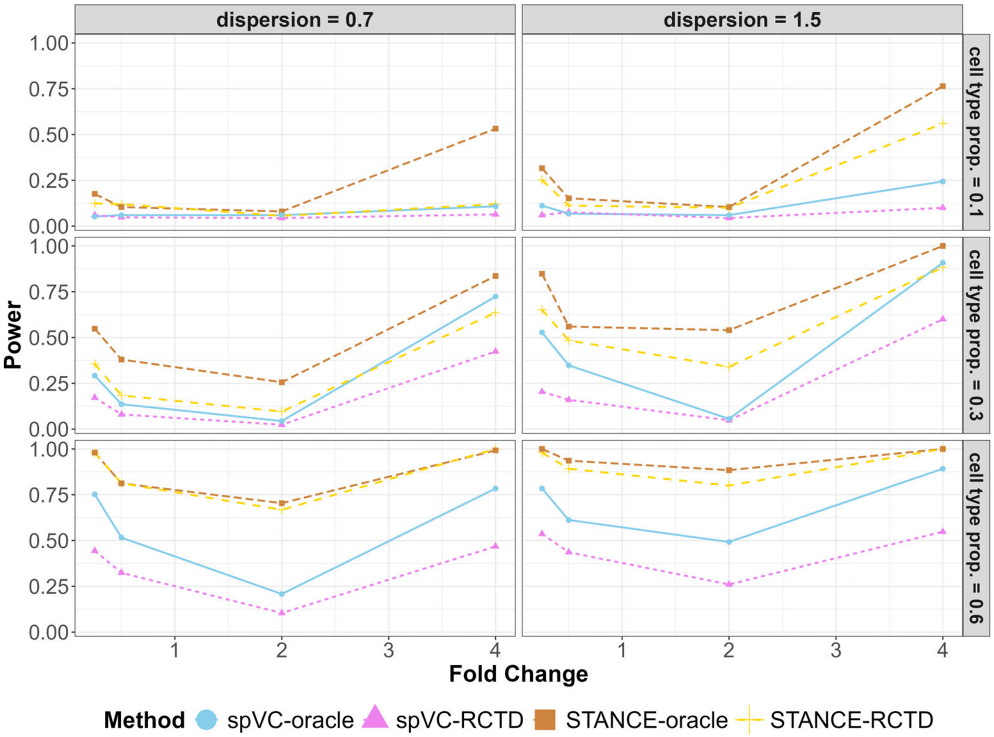

Fig. S9: **Simulation results of the testing power against different fold changes under Simulation 2.** The plots display power values against the fold changes in gene expression for different methods, across various dispersion parameters and cell type proportions, under the significance level of 0.05. Source data are provided as a Source Data file.

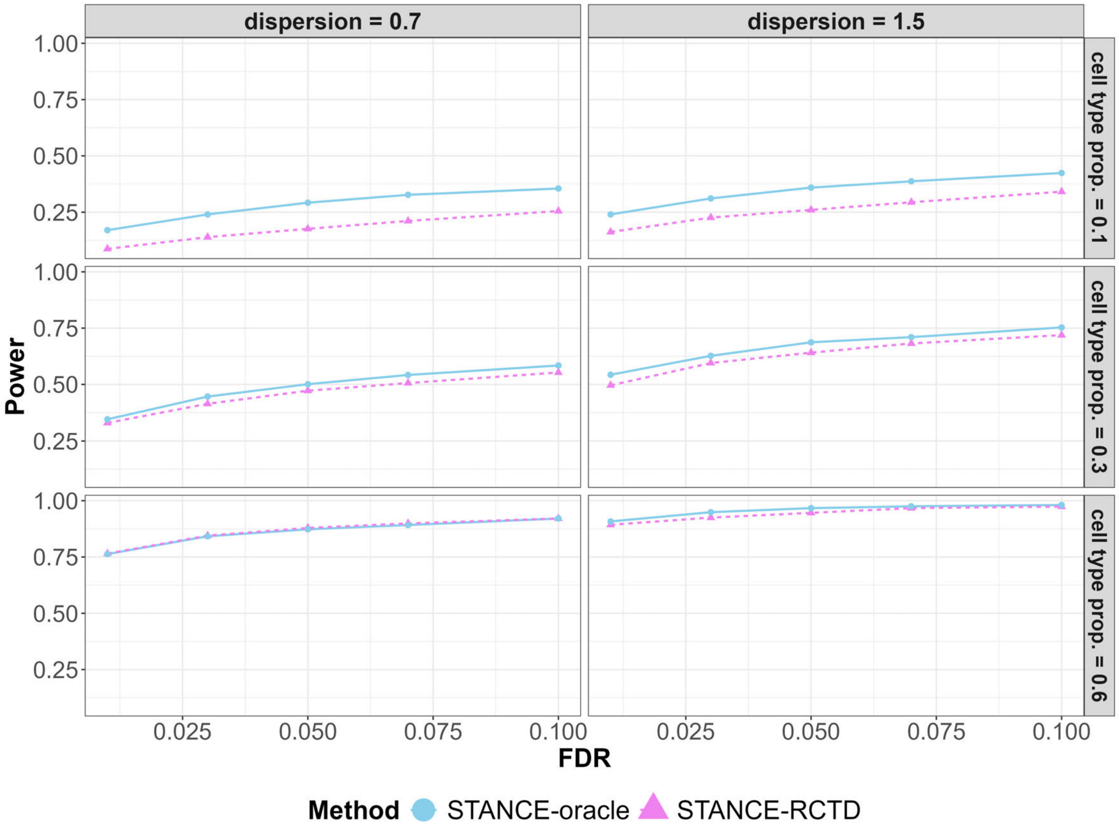

Fig. S10: **Simulation results of the testing power under Simulation 2 (ctSVG vs non-spatial genes ratio: 1/1).** The plots display power values across a range of false discovery rates under the Simulation 2 setting with 300 cell type marker genes, 300 ctSVGs and 300 non-spatial genes. Source data are provided as a Source Data file.

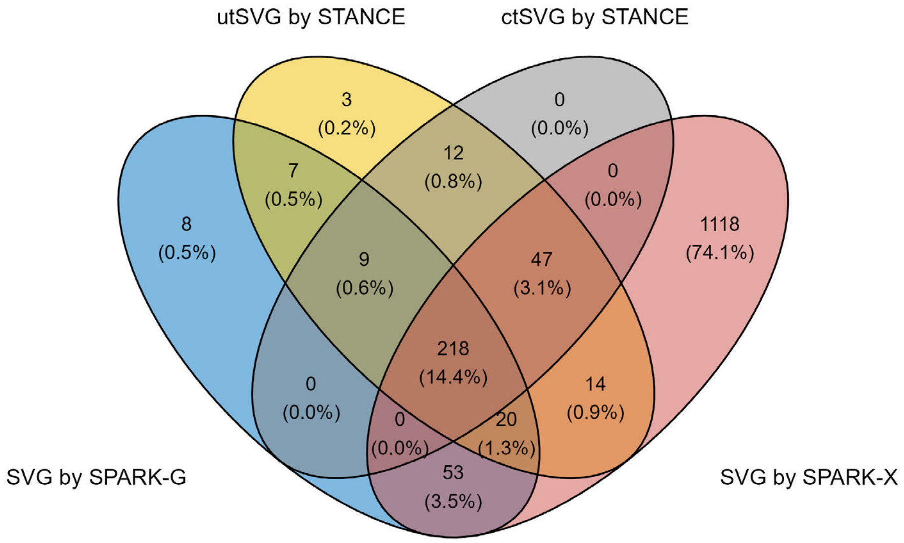

Fig. S11: **The Venn diagram for genes identified by different methods in the human breast cancer dataset.** The Venn diagram shows the logical relationship between sets of genes identified by STANCE, SPARK-G, and SPARK-X. The STANCE overall test identified 330 utSVGs (a mixture of SVGs and ctSVGs), with p-values adjusted by the Benjamini-Yekutieli method with an FDR of 0.05. SPARK-G identified 315 SVGs, of which 254 were also identified by the STANCE overall test. SPARK-X identified 1,470 SVGs, with 299 overlapping with those detected by STANCE. For the 330 utSVGs detected by the STANCE overall test, 286 ctSVGs were identified across all 8 cell types by the STANCE cell-type-specific test. Source data are provided as a Source Data file.

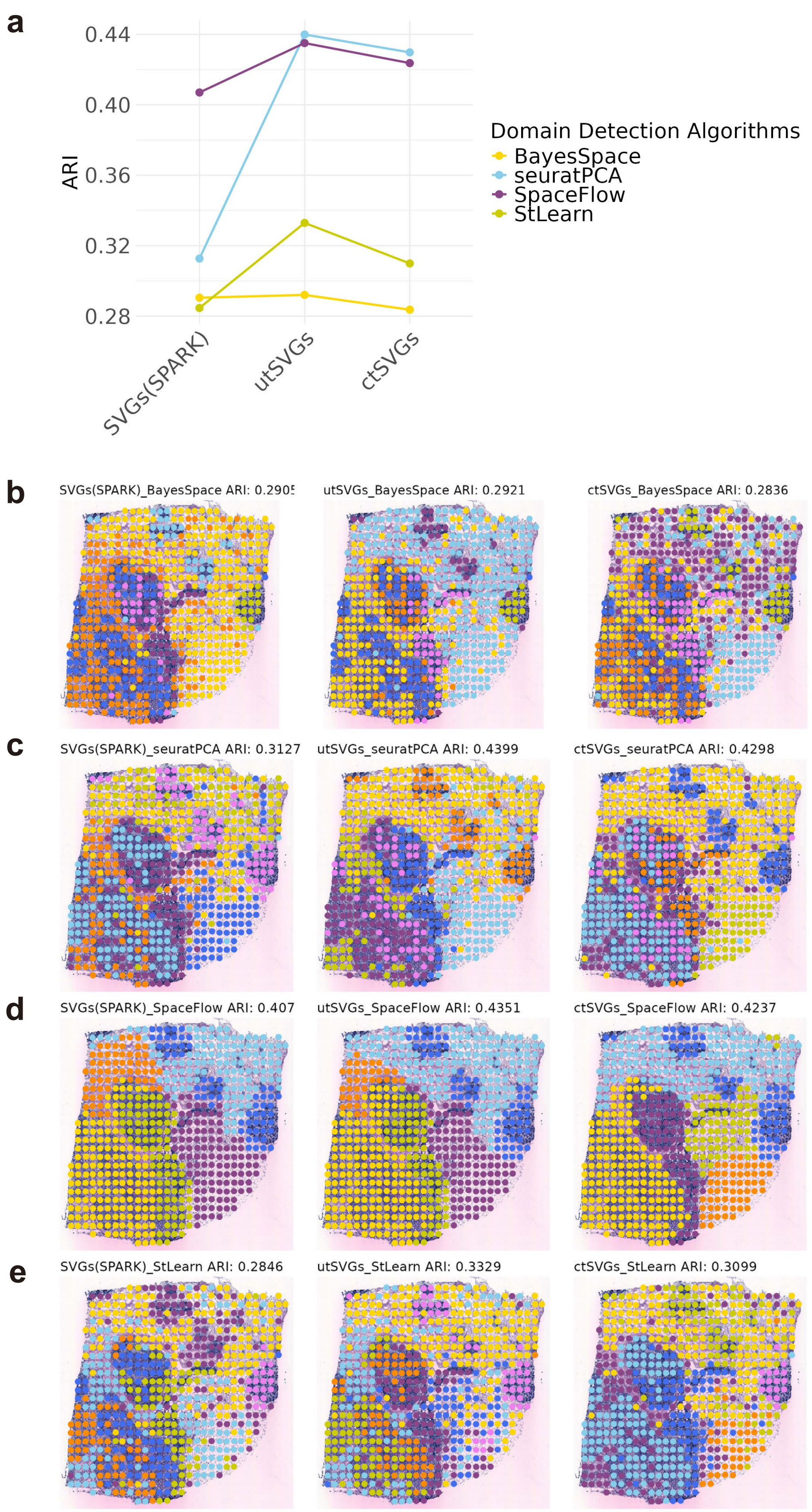
a
0.44
0.40
0.36
0.32
0.28
ARI
SVGs(SPARK)
utSVGs
ctSVGs
Domain Detection Algorithms
BayesSpace
seuratPCA
SpaceFlow
StLearn
b
SVGs(SPARK)_BayesSpace ARI: 0.2905
utSVGs_BayesSpace ARI: 0.2921
ctSVGs_BayesSpace ARI: 0.2836
c
SVGs(SPARK)_seuratPCA ARI: 0.3127
utSVGs_seuratPCA ARI: 0.4399
ctSVGs_seuratPCA ARI: 0.4298
d
SVGs(SPARK)_SpaceFlow ARI: 0.407
utSVGs_SpaceFlow ARI: 0.4351
ctSVGs_SpaceFlow ARI: 0.4237
e
SVGs(SPARK)_StLearn ARI: 0.2846
utSVGs_StLearn ARI: 0.3329
ctSVGs_StLearn ARI: 0.3099

Fig. S12: **Domain detection results illustrating Adjusted Rand Indices (ARIs) and estimated domain annotations obtained using various methods. a.** Comparison of ARIs across four domain detection methods: BayesSpace, SeuratPCA, SpaceFlow, and StLearn. For any given method, using utSVG achieved the highest ARIs, followed by ctSVG and SPARK. This shows the improved domain detection result by STANCE. **b.** Domain annotations estimated by BayesSpace. Left: Results using SVGs identified by SPARK-G. Middle: Results using utSVGs identified by STANCE. Right: Results using ctSVGs identified by STANCE. **c.** Domain annotations estimated by SeuratPCA. Left: Results using SVGs identified by SPARK-G. Middle: Results using utSVGs identified by STANCE. Right: Results using ctSVGs identified by STANCE. **d.** Domain annotations estimated by SpaceFlow. Left: Results using SVGs identified by SPARK-G. Middle: Results using utSVGs identified by STANCE. Right: Results using ctSVGs identified by STANCE. **e.** Domain annotations estimated by StLearn. Left: Results using SVGs identified by SPARK-G. Middle: Results using utSVGs identified by STANCE. Right: Results using ctSVGs identified by STANCE. Source data are provided as a Source Data file.

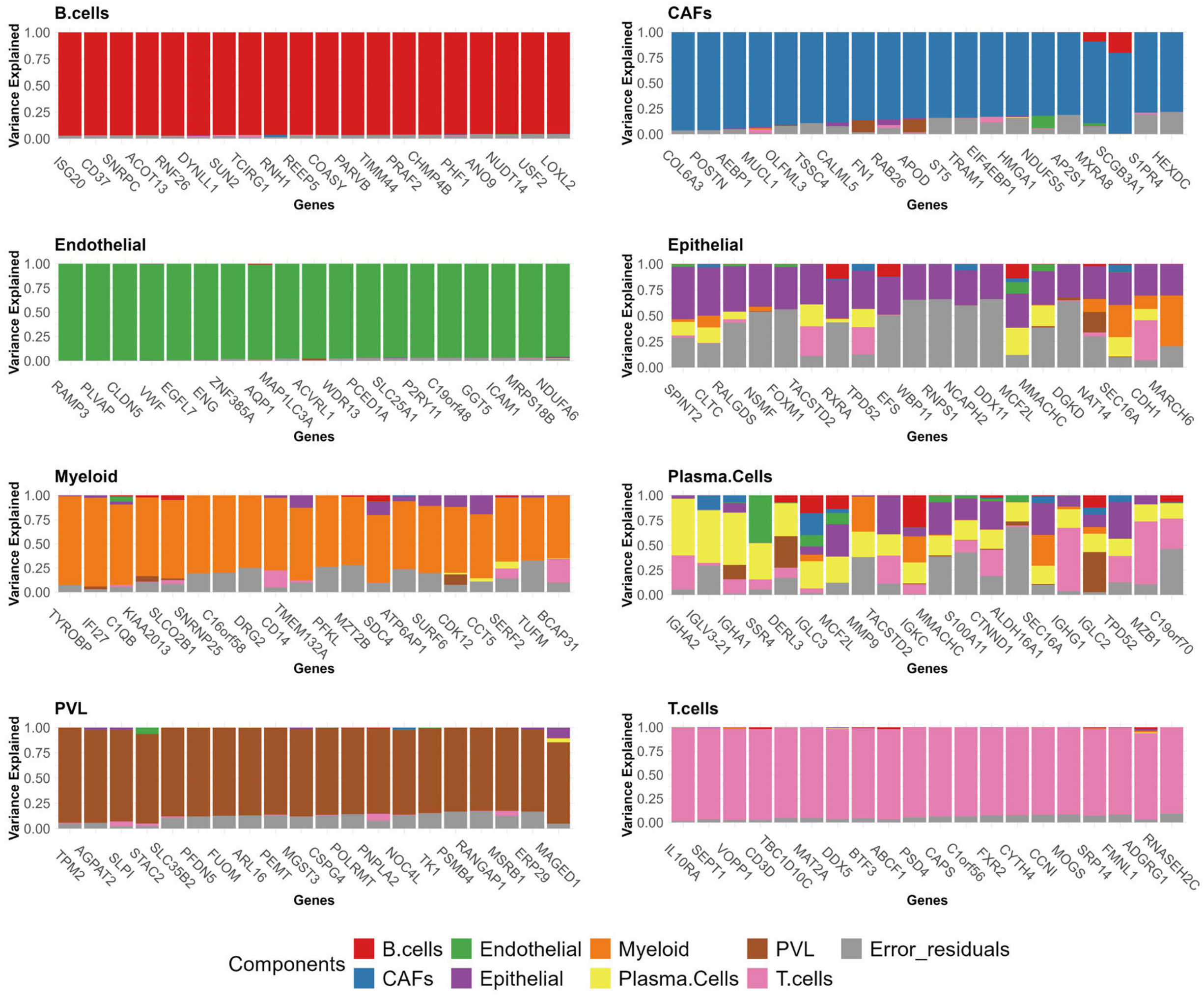

Fig. S13: **The stacked variance plots for each cell type in the human breast cancer dataset.** Displayed are the top 20 significant ctSVGs in each cell type. For each gene, the stacked bar plots display the proportion of variance explained by the 8 cell type-specific spatial effects and random error. Source data are provided as a Source Data file.

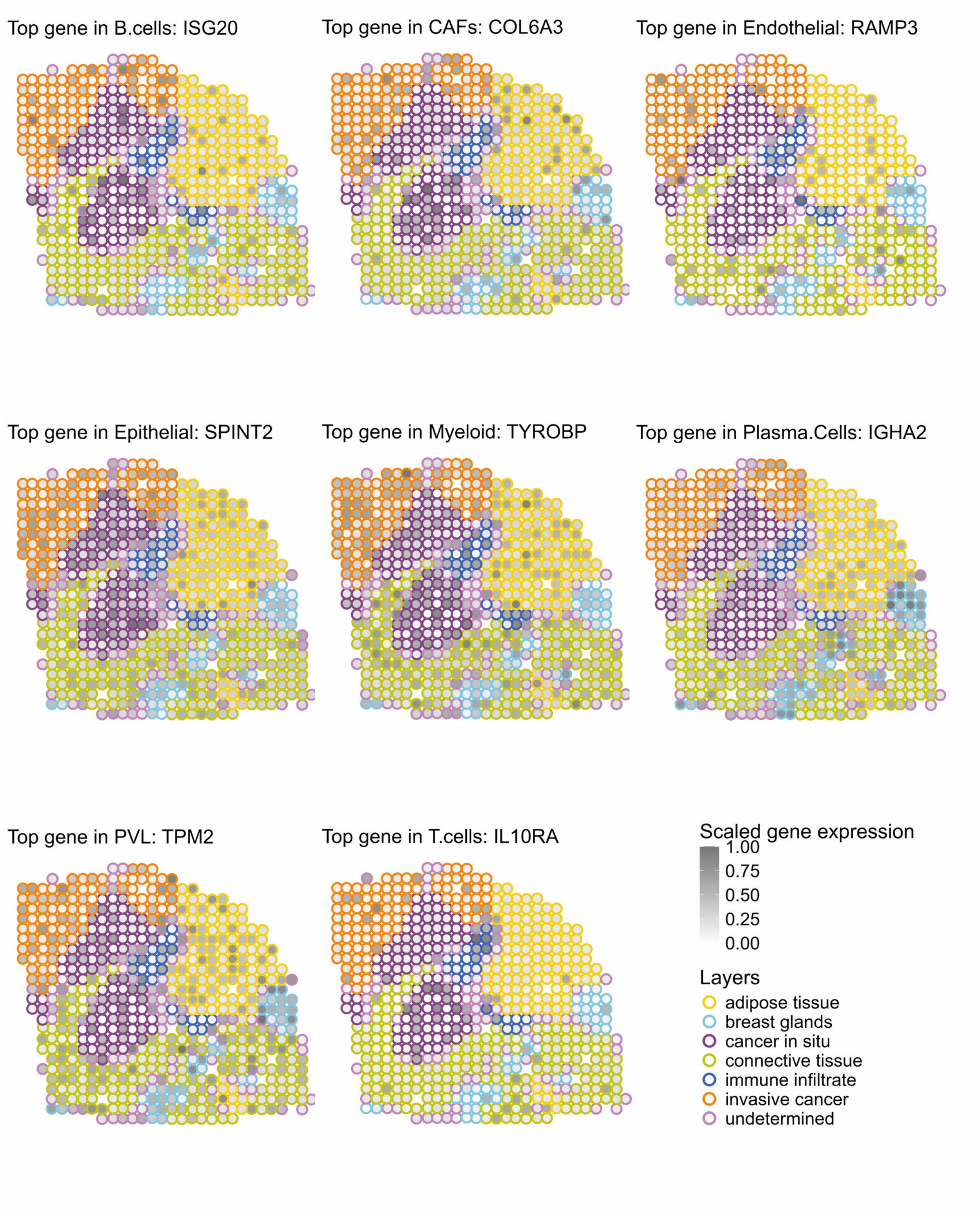

Fig. S14: **Spatial expression pattern plots for the top ctSVGs in each cell type for human breast cancer dataset.** Spots are outlined with colors indicating different annotated domains. The scaled gene expression $\tilde{y}_i = \frac{y_i - \min(\mathbf{y})}{\max(\mathbf{y}) - \min(\mathbf{y})}$ is displayed, where y_i is the original gene expression at spot i.

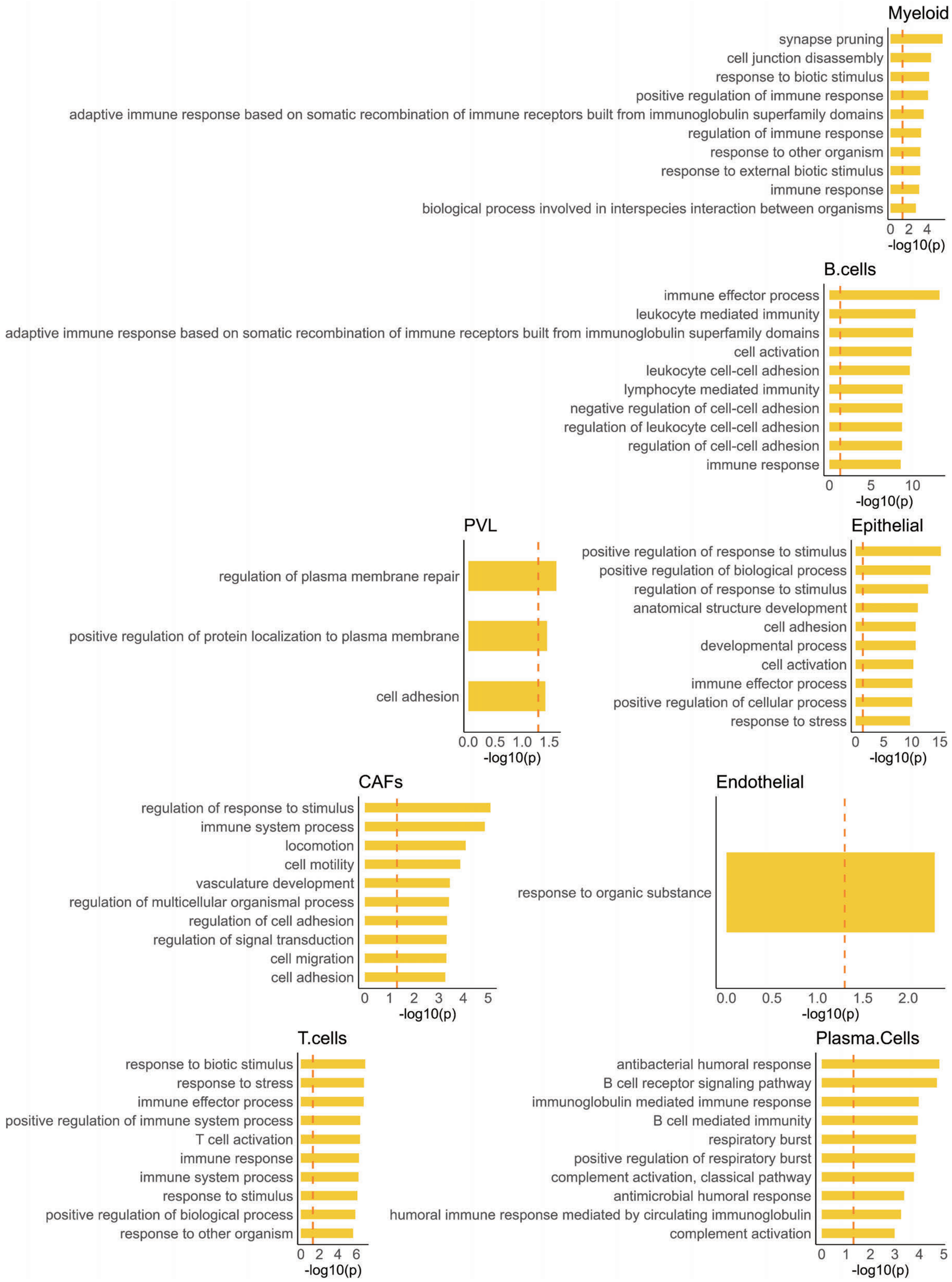

Fig. S15: **The gene set enrichment analysis results for the human breast cancer dataset.** The top 10 significant pathways based on ctSVGs detected by STANCE are shown (if the number of significant pathways is less than 10, then all of them are displayed). The enrichment is given as $-\log_{10}$(adjusted p-value), where the default "g_SCS" algorithm is used for multiple testing corrections. Source data are provided as a Source Data file.

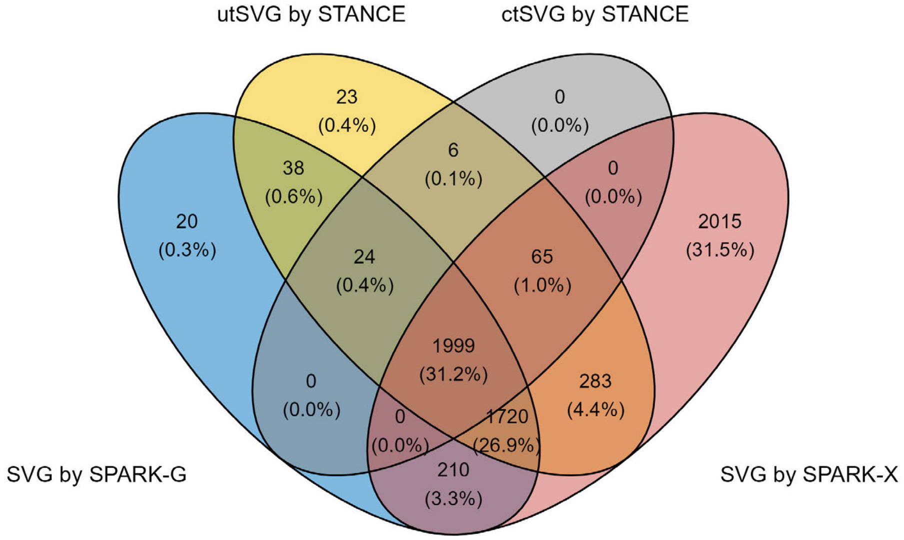

Fig. S16: **The Venn diagram for genes identified by different methods in the human kidney cancer dataset.** The Venn diagram shows the logical relationship between sets of genes identified by STANCE, SPARK-G, and SPARK-X. The STANCE overall test identified 4,158 utSVGs (a mixture of SVGs and ctSVGs), with p-values adjusted by the Benjamini-Yekutieli method to meet an FDR rate of 0.01. SPARK-G identified 4,011 SVGs, among which 3,781 were also identified by the STANCE overall test. SPARK-X identified 6,292 SVGs, with 4,067 overlapping with those detected by STANCE. For the 4,158 utSVGs detected by the STANCE overall test, 2,094 ctSVGs were identified across the 12 cell types. Source data are provided as a Source Data file.

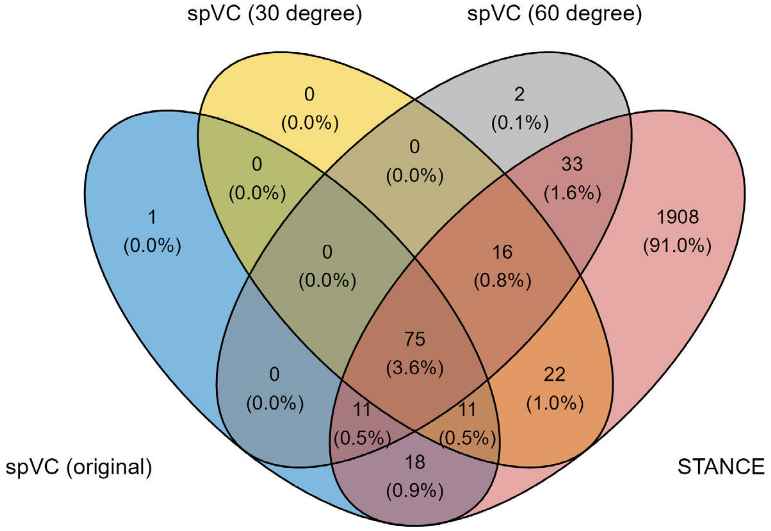

Fig. S17: **The Venn diagram of ctSVGs identified by STANCE and spVC under different spatial rotations in the human kidney cancer dataset.** The Venn diagram illustrates the overlap of ctSVGs identified by STANCE and spVC under various tissue rotations. STANCE identified 2,094 ctSVGs across 12 cell types, with p-values adjusted using the Benjamini-Yekutieli method to control the false discovery rate at 0.01. The same set of ctSVGs was identified by STANCE given its rotation-invariant property. spVC identified 95 ctSVGs under the original tissue pattern, 94 of which overlapped with those identified by STANCE. After a 30° rotation of the tissue, spVC detected 104 ctSVGs, including 86 that overlapped with those from the original pattern. Following a 60° rotation, spVC identified 110 ctSVGs, with 86 shared with the original pattern and 91 overlapping with the 30° rotated pattern. Source data are provided as a Source Data file.

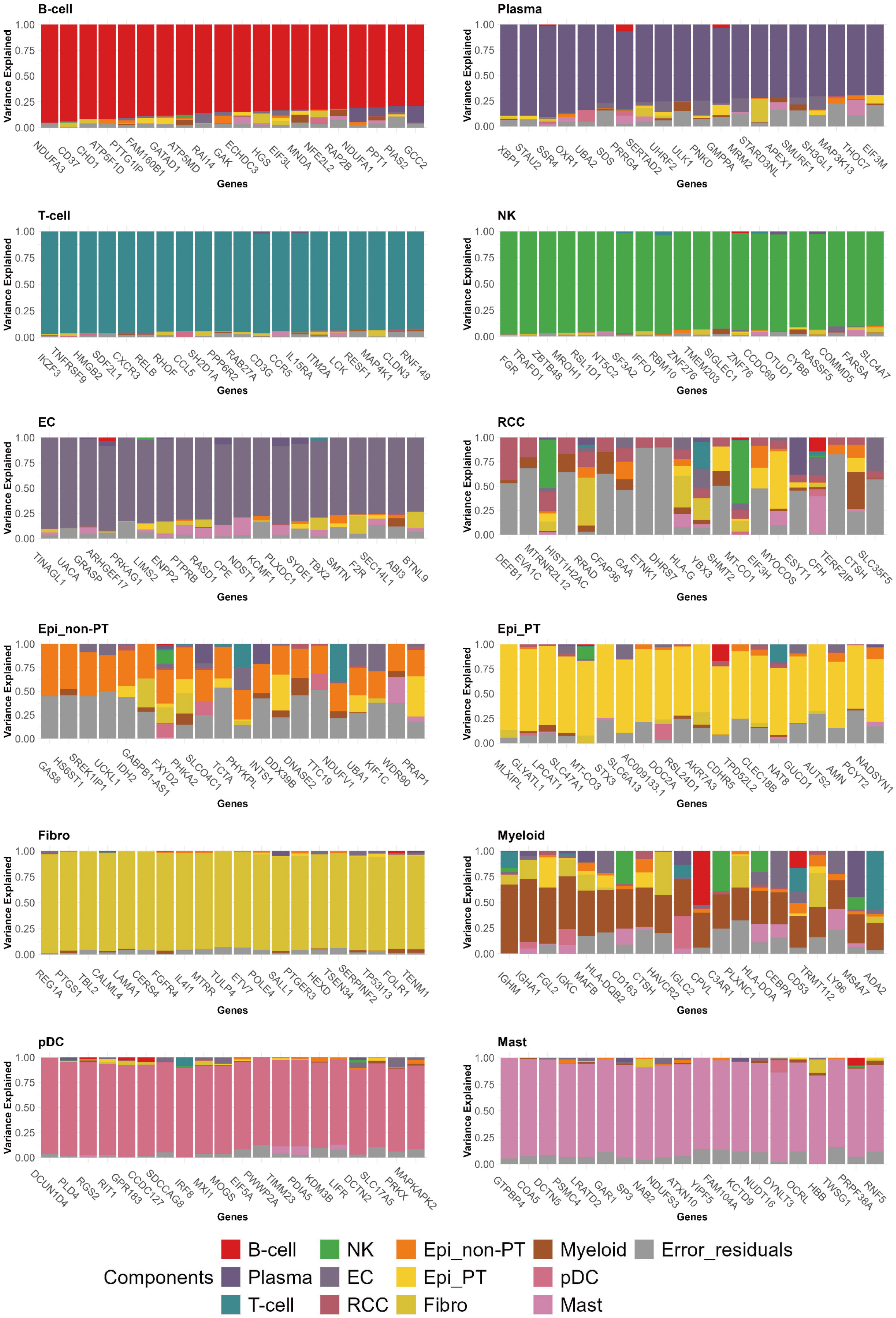
B-cell
Variance Explained
1.00
0.75
0.50
0.25
0.00
NDUFA3 CD37 CHD1 ATP5F1D PTTG1IP FAM160B1 GATAD1 ATP5MD RAI14 GAK ECHDC3 HGS EIF3L MNDA NFE2L2 RAP2B NDUFA1 PPT1 PIAS2 GCC2
Genes
Plasma
XBP1 STAU2 SSR4 OXR1 UBA2 SDS PRRG4 SERTAD2 UHRF2 ULK1 PNKD GMPPA MRM2 STARD3NL APEX1 SMURF1 SH3GL1 MAP3K13 THOC7 EIF3M
T-cell
IKZF3 TNFRSF9 HMGB2 SDF2L1 CXCR3 RELB RHOF CCL5 SH2D1A PPP6R2 RAB27A CD3G CCR5 IL15RA ITM2A LCK RESF1 MAP4K1 CLDN3 RNF149
NK
FGR TRAFD1 ZBTB48 MROH1 RSL1D1 NT5C2 SF3A2 IFFO1 RBM10 ZNF276 TMEM203 SIGLEC1 ZNF76 CCDC69 OTUD1 CYBB RASSF5 COMMD5 FARSA SLC4A7
EC
TINAGL1 UACA GRASP ARHGEF17 PRKAG1 LIMS2 ENPP2 PTPRB RASD1 CPE NDST1 KCMF1 PLXDC1 SYDE1 TBX2 SMTN F2R SEC14L1 ABI3 BTNL9
RCC
DEFB1 EVA1C MTRNR2L12 HIST1H2AC RRAD CFAP36 GAA ETNK1 DHRS7 HLA-G YBX3 SHMT2 MT-CO1 EIF3H MYOCOS ESYT1 CFH TERF2IP CTSH SLC35F5
Epi_non-PT
GAS8 HS6ST1 SREK1IP1 UCKL1 IDH2 GABPB1-AS1 FXYD2 PHKA2 SLCO4C1 TCTA PHYKPL INTS1 DDX39B DNASE2 TTC19 NDUFV1 UBA1 KIF1C WDR90 PRAP1
Epi_PT
MLXIPL GLYATL1 LPCAT1 SLC47A1 MT-CO3 STX3 SLC6A13 AC009133.1 DOC2A RSL24D1 AKR7A3 CDHR5 TPD52L2 CLEC18B NAT8 GUCD1 AUTS2 AMN PCYT2 NADSYN1
Fibro
REG1A PTGS1 TBL2 CALML4 LAMA1 CERS4 FGFR4 IL4I1 MTRR TULP4 ETV7 POLE4 SALL1 PTGER3 HEXD TSEN34 SERPINF2 TP53I13 FOLR1 TENM1
Myeloid
IGHM IGHA1 FGL2 IGKC MAFB HLA-DQB2 CD163 CTSH HAVCR2 IGLC2 CPVL C3AR1 PLXNC1 HLA-DOA CEBPA CD53 TRMT112 LY96 MS4A7 ADA2
pDC
DCUN1D4 PLD4 RGS2 RIT1 GPR183 CCDC127 SDCCAG8 IRF8 MXI1 MOGS EIF5A PWWP2A TIMM23 PDIA5 KDM3B LIFR DCTN2 SLC17A5 PRKX MAPKAPK2
Mast
GTPBP4 COA5 DCTN5 PSMC4 LRATD2 GAR1 SP3 NAB2 NDUFS3 ATXN10 YIPF5 FAM104A KCTD9 NUDT16 DYNLT3 OCRL HBB TWSG1 PRPF38A RNF5
Components
B-cell
Plasma
T-cell
NK
EC
RCC
Epi_non-PT
Epi_PT
Fibro
Myeloid
pDC
Mast
Error_residuals

Fig. S18: **The stacked variance plots for each cell type in the human kidney cancer dataset.** Displayed are the top 20 significant ctSVGs in each cell type. For each gene, the stacked bar plots display the proportion of variance explained by the 12 cell-type-specific spatial effects and random error. The stacked bar plots indicate that the top 20 ctSVGs of renal cell carcinoma (RCC) cells, non-proximal tubule epithelial (Epi_non-PT) cells and myeloid cells are more heterogeneous in variance than those of the other cell types. Source data are provided as a Source Data file.

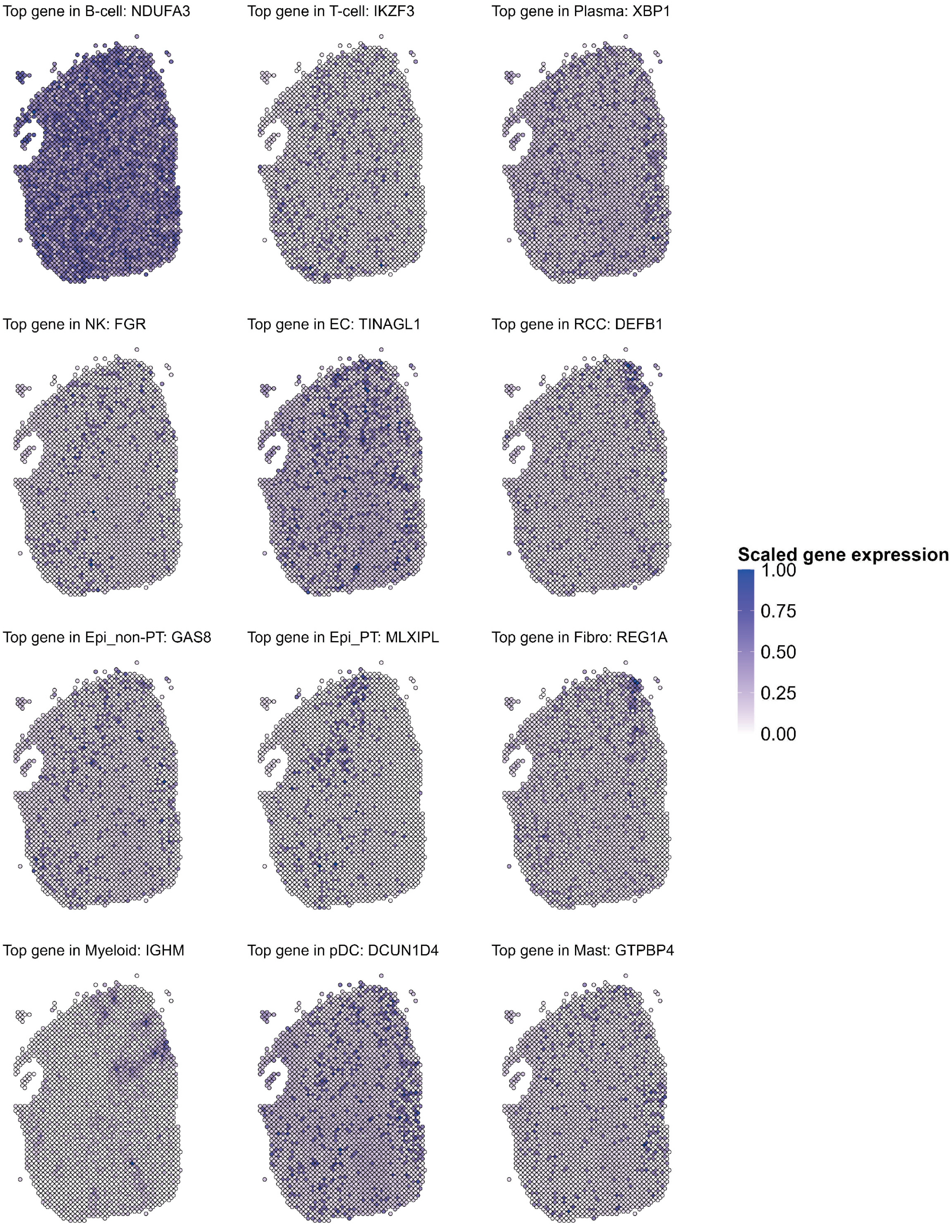

Fig. S19: **Spatial pattern plots for the top ctSVGs of each cell type in the human kidney dataset.** The scaled gene expression $\tilde{y}_i = \frac{y_i - \min(\mathbf{y})}{\max(\mathbf{y}) - \min(\mathbf{y})}$ is displayed, where y_i is the original gene expression at spot i.

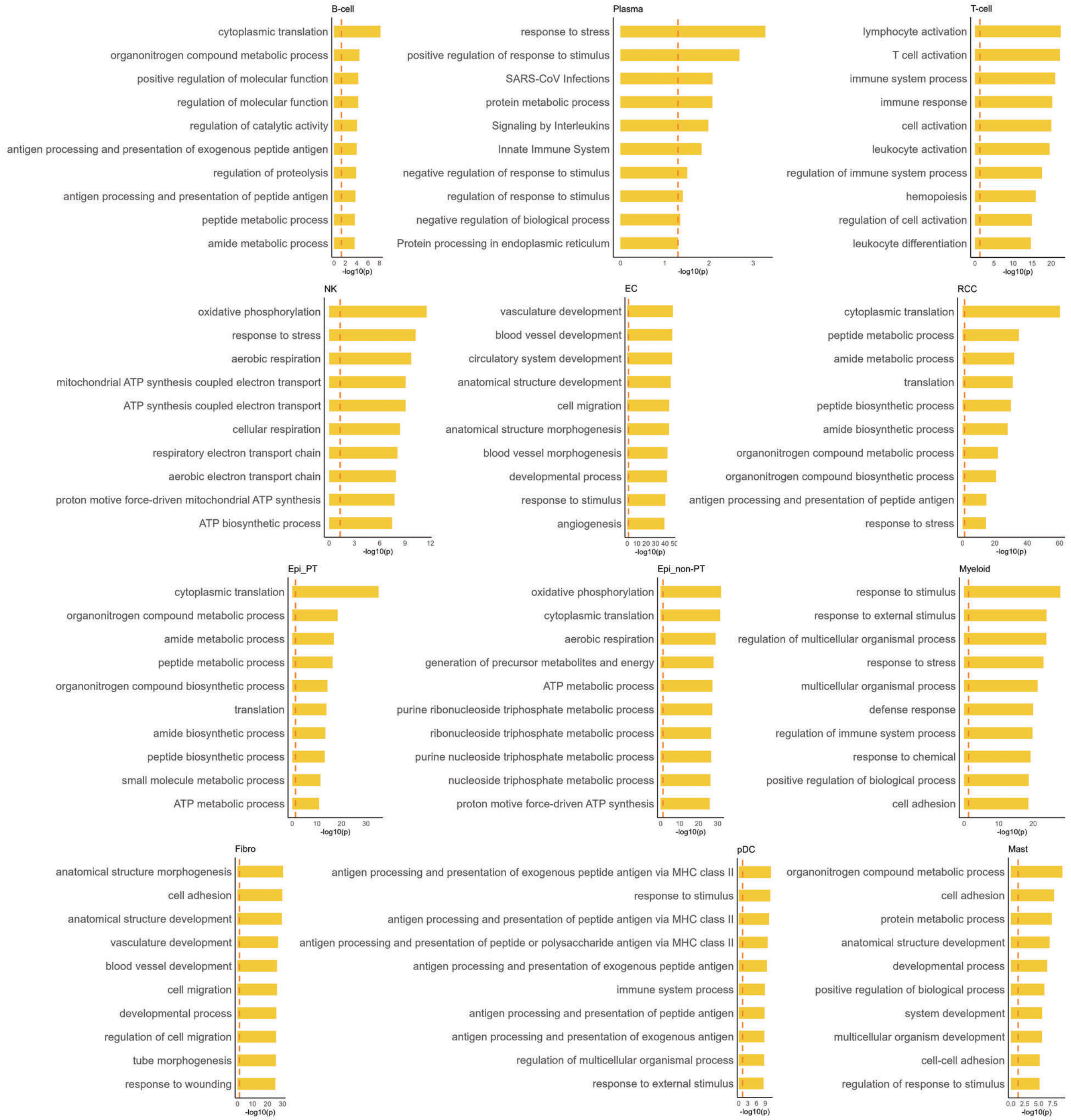

Fig. S20: **The gene set enrichment analysis results for the human kidney cancer dataset.** The top 10 significant pathways based on ctSVGs detected by STANCE are shown (if the number of significant pathways is less than 10, then all of them are displayed). The enrichment is given as $-\log_{10}$(adjusted p-value), where the default "g_SCS" algorithm is used for multiple testing corrections. Source data are provided as a Source Data file.

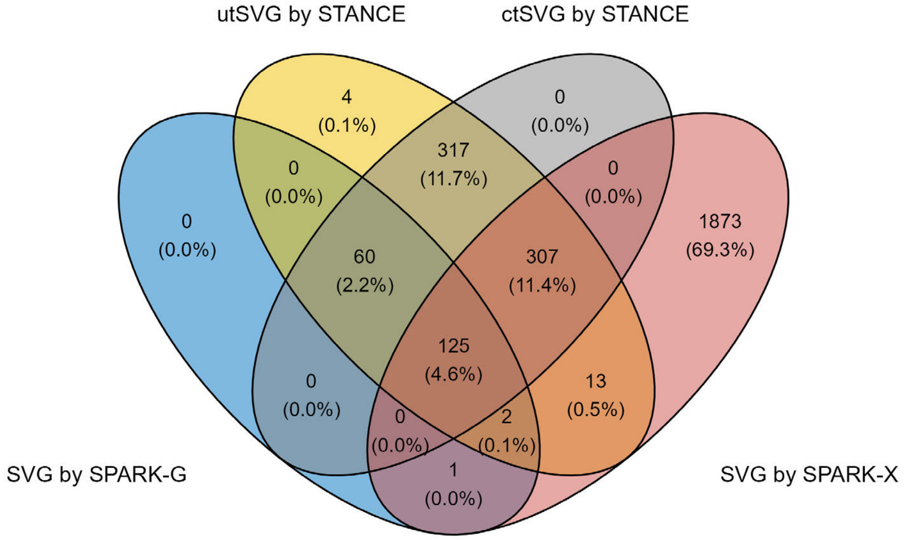

Fig. S21: **The Venn diagram for genes identified by different methods in the mouse olfactory bulb dataset.** The Venn diagram shows the logical relationship between sets of genes identified by STANCE, SPARK-G, and SPARK-X. The STANCE overall test identified 828 utSVGs (a mixture of SVGs and ctSVGs), with p-values adjusted by the Benjamini-Yekutieli method to meet an FDR rate of 0.05. SPARK-G identified 188 SVGs, 187 of which were also identified by the STANCE overall test. SPARK-X identified 2,321 SVGs, with 447 overlapping with those detected by STANCE. For the 828 utSVGs detected by the STANCE overall test, 809 ctSVGs were identified across the 12 cell types. Source data are provided as a Source Data file.

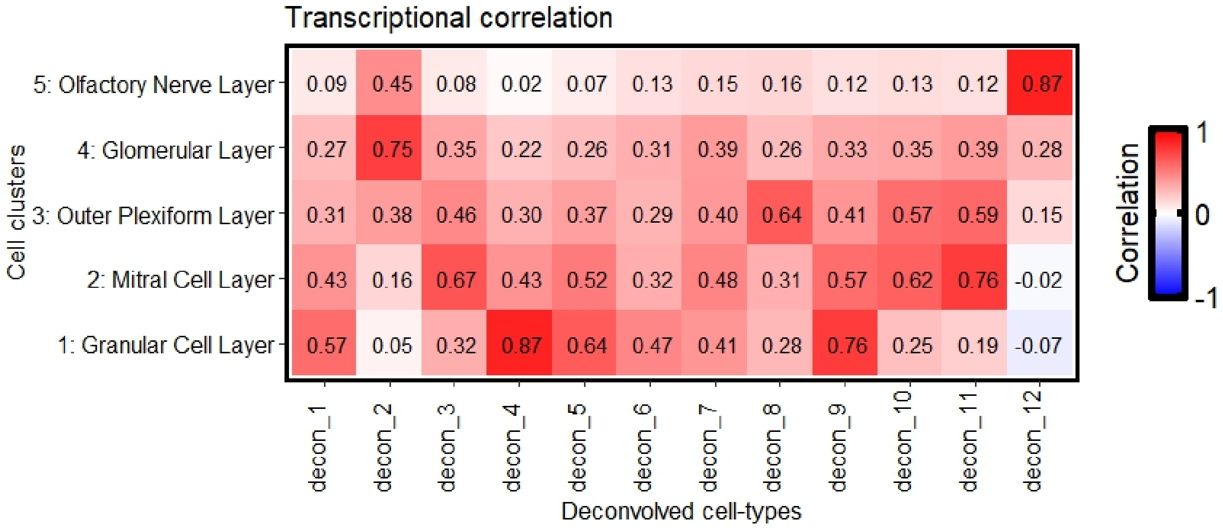

Fig. S22: **The transcriptional correlation heatmap for deconvolved cell types and cell clusters in mouse olfactory bulb dataset.** The heatmap visualizes the transcriptional correlation between deconvolved cell types and cell clusters (layers). Specifically, deconvolved cell type 4, cell type 11, cell type 8, cell type 2 and cell type 12 are highly expressed in grangular cell layer, mitral cell layer, outer plexiform layer, glomerular layer and olfactory nerve layer respectively, with highest correlation. Source data are provided as a Source Data file.

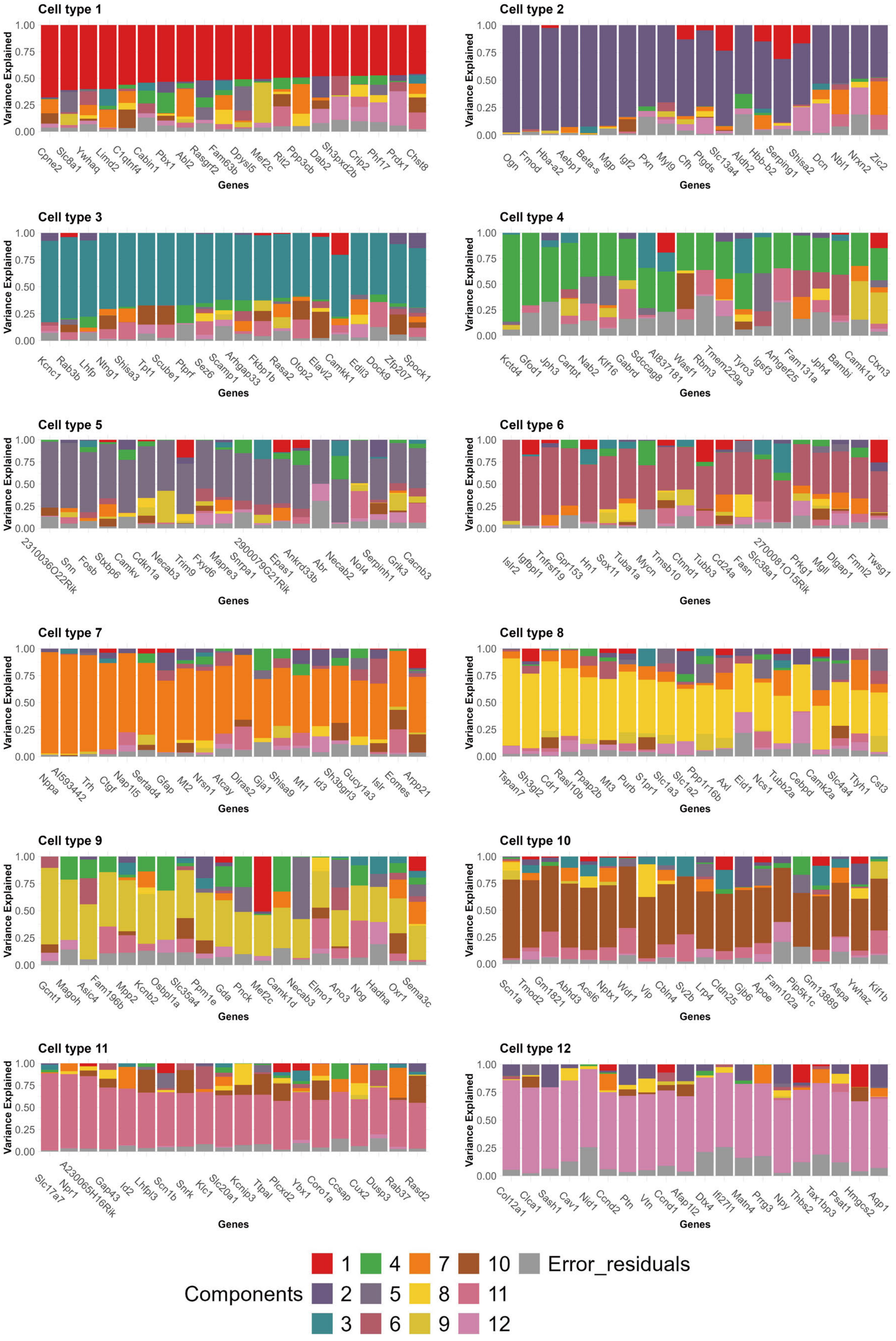
Cell type 1
Variance Explained
1.00 0.75 0.50 0.25 0.00
Cpne2 Slc8a1 Ywhaq Lmd2 C1qtnf4 Cabin1 Pbx1 Abl2 Rasgrf2 Fam63b Dpysl5 Mef2c Rit2 Ppp3cb Dab2 Sh3pxd2b Crip2 Phf17 Prdx1 Chst8
Genes
Cell type 2
Ogn Fmod Hba-a2 Aebp1 Beta-s Mgp Igf2 Pxn Myl9 Cfh Ptgds Slc13a4 Aldh2 Hbb-b2 Serping1 Shisa2 Dcn Nbl1 Nrxn2 Zic2
Cell type 3
Kcnc1 Rab3b Lhfp Ntng1 Shisa3 Tpt1 Scube1 Ptprf Sez6 Scamp1 Arhgap33 Fkbp1b Rasa2 Otop2 Elavl2 Camkk1 Edil3 Dock9 Zfp207 Spock1
Cell type 4
Kctd4 Gfod1 Jph3 Cartpt Nab2 Klf16 Gabrd Sdccag8 AI837181 Wasf1 Rbm3 Tmem229a Tyro3 Igsf3 Arhgef25 Fam131a Jph4 Bambi Camk1d Ctxn3
Cell type 5
2310036O22Rik Snn Fosb Stxbp6 Camkv Cdkn1a Necab3 Trim9 Fxyd6 Mapre3 Snrpa1 2900079G21Rik Epas1 Ankrd33b Abr Necab2 Nol4 Serpinh1 Grik3 Cacnb3
Cell type 6
Islr2 Igfbpl1 Tnfrsf19 Gpr153 Hn1 Sox11 Tuba1a Mycn Tmsb10 Ctnnd1 Tubb3 Cd24a Fasn Slc38a1 2700081O15Rik Prkg1 Mgll Dlgap1 Fmnl2 Twsg1
Cell type 7
Nppa AI593442 Trh Ctgf Nap1l5 Sertad4 Gfap Mt2 Nrsn1 Atcay Diras2 Gja1 Shisa9 Mt1 Id3 Sh3bgrl3 Gucy1a3 Islr Eomes Arpp21
Cell type 8
Tspan7 Sh3gl2 Cdr1 Rasl10b Ppap2b Mt3 Purb S1pr1 Slc1a3 Slc1a2 Ppp1r16b Axl Eid1 Ncs1 Tubb2a Cebpd Camk2a Slc4a4 Ttyh1 Cst3
Cell type 9
Gcnt1 Magoh Asic4 Fam196b Mpp2 Kcnb2 Osbpl1a Slc35a4 Ppm1e Gda Pnck Mef2c Camk1d Necab3 Elmo1 Ano3 Nog Hadha Oxr1 Sema3c
Cell type 10
Scn1a Tmod2 Gm1821 Abhd3 Acsl6 Nptx1 Wdr1 Vip Cbln4 Sv2b Lrp4 Cldn25 Gjb6 Apoe Fam102a Pip5k1c Gm13889 Aspa Ywhaz Kif1b
Cell type 11
Slc17a7 Npr1 A230065H16Rik Gap43 Id2 Lhfpl3 Scn1b Snrk Klc1 Slc20a1 Kcnip3 Tpbg Plcxd2 Ybx1 Coro1a Ccsap Cux2 Dusp3 Rab37 Rasd2
Cell type 12
Col12a1 Clca1 Sash1 Cav1 Nid1 Ccnd2 Ptn Vtn Ccnd1 Afap1l2 Dtx4 Ifi27l1 Matn4 Prrg3 Npy Thbs2 Tax1bp3 Psat1 Hmgcs2 Aqp1
Components
1 2 3 4 5 6 7 8 9 10 11 12 Error_residuals

Fig. S23: **The stacked variance plots for each cell type in the mouse olfactory bulb dataset.** Displayed are the top 20 significant ctSVGs in each cell type. For each gene, the stacked bar plots display the proportion of variance explained by the 12 cell-type-specific spatial effects and random error. Source data are provided as a Source Data file.

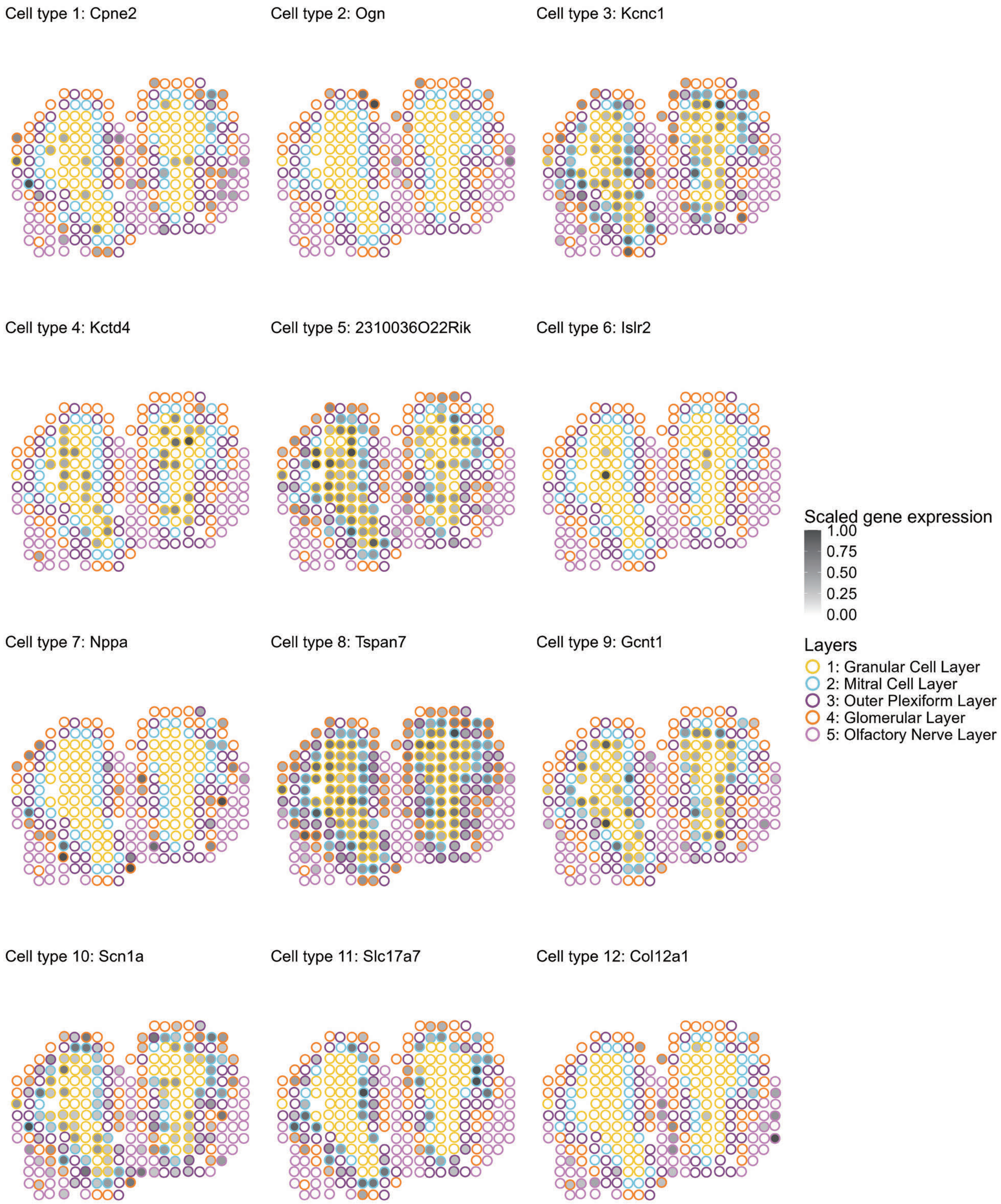

Fig. S24: **Spatial pattern plots for the top ctSVGs of each cell type in the mouse olfactory bulb dataset.** Spots are outlined with colors indicating the annotated domains. The scaled gene expression $\tilde{y}_i = \frac{y_i - \min(\mathbf{y})}{\max(\mathbf{y}) - \min(\mathbf{y})}$ is displayed, where y_i is the original gene expression at spot i.

Spatial Rotation Simulation

We did a simulation to demonstrate that CSIDE, spVC and CTSV have statistical issues in ctSVG detection, due to the fact that they treat spatial locations as fixed effect and such analysis are not spatial rotation-invariant.

Import packages and the sample pattern

```
require(ggplot2)
require(dplyr)
require(tidyr)
if (!require("devtools", quietly = TRUE)){
  install.packages("devtools")
}
if (!requireNamespace("BiocManager", quietly = TRUE)){
  install.packages("BiocManager")
}
#devtools::install_github("dmcable/spacexr", build_vignettes = FALSE)
require(spacexr)
#BiocManager::install("SpatialExperiment")
#BiocManager::install("CTSV", version = "devel")
require(SpatialExperiment)
require(CTSV)
#devtools::install_github("funstatpackages/Triangulation")
require(Triangulation)
#devtools::install_github("shanyu-stat/spVC")
require(spVC)
#devtools::install_github("Cui-STT-Lab/STANCE")
require(STANCE)
```

Simulate single cell resolution spatial transcriptomics data

We first simulated single cell resolution spatial transcriptomics data. We borrowed the tissue shape of the mouse olfactory bulb (MOB) dataset [20] and generated 3000 single cells as well as 3 spatial domains through the `SRTsim`[24] package. (Here, the pre-simulated single cell data `Sample.rda` is available at https://drive.google.com/drive/folders/1KSxeInbwFswuJdTBxCjMZKc6voz5UWqZ?usp=drive_link.)

```
set.seed(1)
load(file = "./Sample.rda")
# number of single cells
numCells <- nrow(dat.sc)

plot_spatial_pattern <- ggplot(dat.sc, aes(x, y, color = domain)) +
```

```
  geom_point() +
  theme_minimal() +
  theme(legend.title = element_text(size = 11, face = "bold"),
        panel.grid.major = element_blank(),
        panel.grid.minor = element_blank()) +
  labs(color = "Domain") +
  guides(color = guide_legend(override.aes = list(size = 4)))

print(plot_spatial_pattern)
```

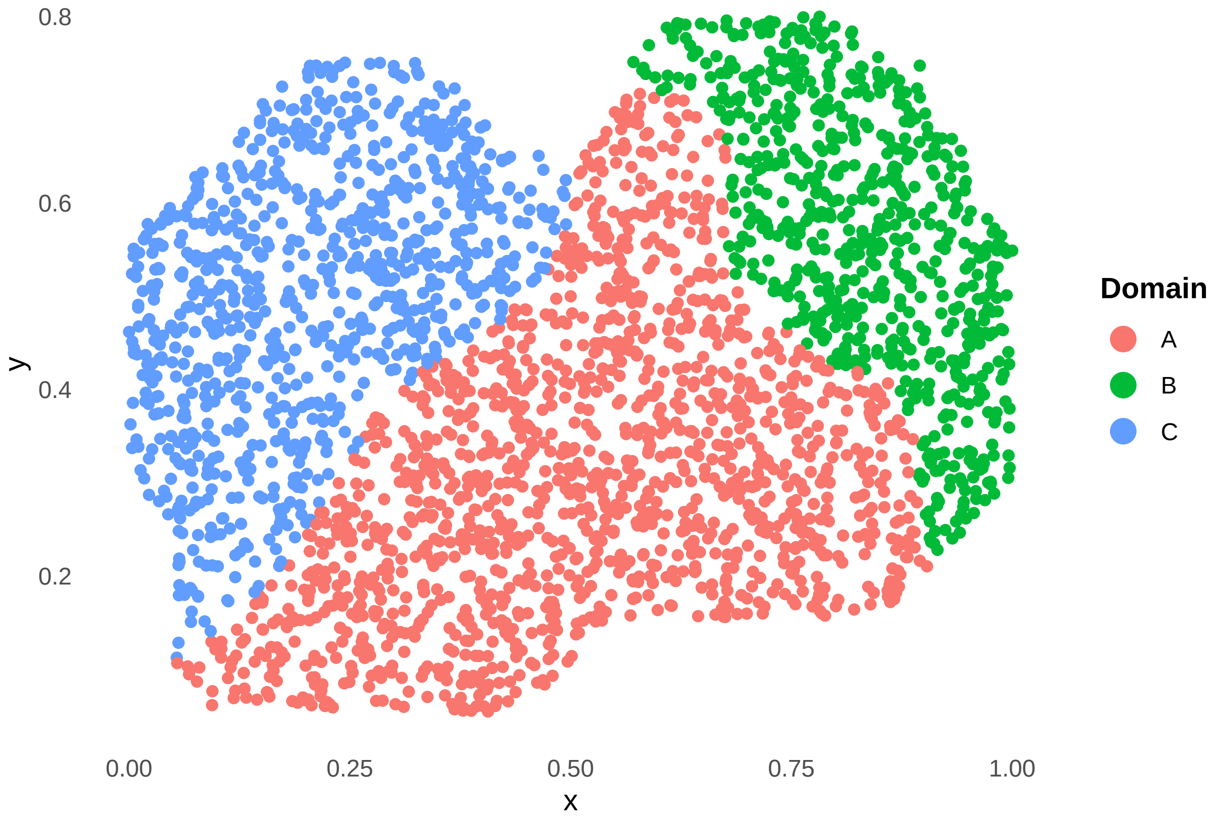

Each cell is assigned to one of three cell types based on a categorical distribution, with probabilities of 40% for cell type 1 (CT1), 30% for cell type 2 (CT2), and 30% for cell type 3 (CT3).

```
# Assign single cells into 3 cell type groups
Cell_Types <- paste0("CT",extraDistr::rcat(n = numCells,
                                          prob = c(0.4, 0.3, 0.3)))
dat.sc$cell_type <- factor(Cell_Types)
plot_CT_pattern <- ggplot(dat.sc, aes(x, y, color = cell_type)) +
  geom_point() +
  theme_minimal() +
  theme(legend.title = element_text(size = 11, face = "bold"),
        panel.grid.major = element_blank(),
        panel.grid.minor = element_blank()) +
  labs(color = "Cell types") +
```

```r
  guides(color = guide_legend(override.aes = list(size = 4)))
print(plot_CT_pattern)
```

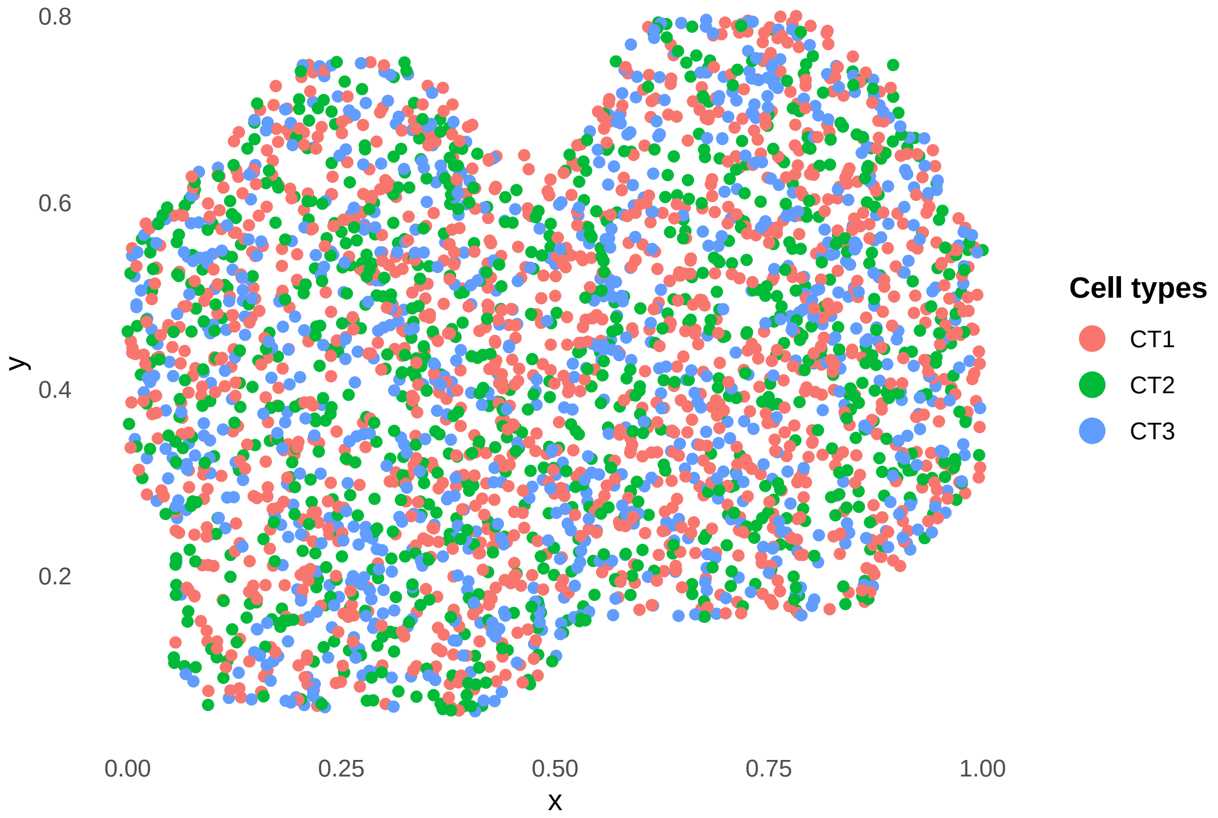

We assumed the presence of 3 distinct cell types and simulated the expression of 50 genes per cell using a negative binomial distributions characterized by mean 1 and dispersion parameter 1.5.

```r
# number of genes
numGenes <- 50
# mean and dispersion parameter of negative binomial distribution
mu <- 1
dispersion <- 0.7
# Baseline expression
counts.null <- matrix(rnbinom(n = (numGenes * numCells),
                              size = dispersion,
                              mu = mu),nrow = numGenes)
counts.sc <- counts.null
```

We selected 30 out of 50 genes serving as cell type marker genes, in which each of three cell types has 10 unique marker genes. For each specific cell type, we modified the expression of their marker genes with a fold change of 4 (i.e., multiplying the mean parameter of the negative binomial distribution by 4), regardless of their domain assignments.

```r
# Fold change the mean by 4 to the expression of 10 marker genes for each cell type
for (iCT in 1:3){
```

```
  counts.upregulated <- matrix(rnbinom(n = (10 * numCells),
                                       size = dispersion,
                                       mu = mu * 4),nrow = 10)
  cell_fold_change.1 <- which((dat.sc$cell_type == paste0("CT",iCT)))
  counts.sc[(iCT*10 - 9):(iCT*10),
            cell_fold_change.1] <- counts.upregulated[,cell_fold_change.1]
}
```

Next, we selected another 10 genes, Gene 31 to Gene 40, to serve as CT1-specific ctSVGs. For cell type CT1, we adjust the mean expression of the genes with a fold change of 4 for cells located outside domain A.

```
cell_fold_change.2 <- which((dat.sc$domain != "A") & (dat.sc$cell_type == "CT1"))
# Fold change the mean by 4 to the expression of 10 SVGs
# for the single cells within domain B & C
counts.sc[31:40,cell_fold_change.2] <- matrix(
  rnbinom(n = (10 * length(cell_fold_change.2)),size = dispersion,mu = mu * 4),
  nrow = 10)

row.names(counts.sc) <- paste0("Gene", 1:dim(counts.sc)[1])
colnames(counts.sc) <- paste0("Cell", 1:dim(counts.sc)[2])
```

The tissue was divided into 251 spots using a grid size of 0.05. For each spot, we aggregated the expression counts of all cells within it to determine spot-level expression and calculated cell type compositions. Additionally, the coordinates of each spot were based on the mean x and y coordinates of the cells within that spot.

```
## Aggregate to obtain spot-level gene expression
counts.st <- apply(counts.sc, MARGIN = 1,
                   FUN = function(gene_expr, dat = dat.sc, grid.size = 0.05){
                     # Combine gene expression vector into dat
                     dat <- cbind(dat, gene_expr)

                     # Calculate grid indices for each cell
                     dat$grid_x <- floor(dat$x / grid.size)
                     dat$grid_y <- floor(dat$y / grid.size)

                     # Aggregate gene expression by grid and calculate center of mass
                     expression_summary <- dat %>%
                       group_by(grid_x, grid_y) %>%
                       summarise(
                         expr = sum(gene_expr),
                         .groups = 'drop'
                       )
                     return(expression_summary$expr)
                   })
counts.st <- t(counts.st)

# number of spots
n <- ncol(counts.st)

# Calculate proportions of each cell type within each grid
cell_type_counts <- dat.sc %>%
```

```r
  group_by(grid_x, grid_y, cell_type) %>%
  summarise(count = n(),
            #            x_center = mean(x),
            #            y_center = mean(y),
            .groups = 'drop')

total_counts <- dat.sc %>%
  group_by(grid_x, grid_y) %>%
  summarise(total = n(), .groups = 'drop')

proportions <- cell_type_counts %>%
  left_join(total_counts, by = c("grid_x", "grid_y")) %>%
  mutate(proportion = count / total) %>%
  select(grid_x, grid_y, cell_type, proportion)

# Pivot the proportions table for each cell type into columns
proportions_wide <- proportions %>%
  pivot_wider(names_from = cell_type,
              values_from = proportion,
              values_fill = list(proportion = 0))

# Get spot-level data frame
dat.st <- expression_summary <- dat.sc %>%
  group_by(grid_x, grid_y) %>%
  summarise(
    x_center = mean(x),
    y_center = mean(y),
    .groups = 'drop'
  ) %>%
  left_join(proportions_wide, by = c("grid_x", "grid_y")) %>%
  select(x_center, y_center, CT1, CT2, CT3)

# Coordinates matrix
pos.original <- dat.st %>%
  select(x = x_center, y = y_center)
pos.original <- as.matrix(pos.original)
row.names(pos.original) <- paste0("Spot", 1:dim(pos.original)[1])

# Proportion matrix
prop <- dat.st %>%
  select(-c(x_center, y_center))
prop <- as.matrix(prop)
row.names(prop) <- paste0("Spot", 1:dim(prop)[1])

# Expression count matrix
counts <- counts.st
row.names(counts) <- paste0("Gene", 1:dim(counts)[1])
colnames(counts) <- paste0("Spot", 1:dim(counts)[2])
```

Rotate the tissue

Define the `rotate_points` function.

```r
rotate_points <- function(original_points, angle_degrees) {
  angle_radians <- angle_degrees * (pi / 180)  # Convert degrees to radians
  rotation_matrix <- matrix(c(cos(angle_radians), -sin(angle_radians),
                              sin(angle_radians),  cos(angle_radians)),
                            nrow = 2, byrow = TRUE)
  rotated_points <- t(rotation_matrix %*% t(original_points))
  output <- as.matrix(rotated_points)
  colnames(output) <- c('x', 'y')
  return(output)
}
```

Set the rotation angles to be 30°, 60° and 90°, rotate the tissue and then scale the new x and y coordinates.

```r
# Rotate the original pattern by the given angle degree
pos.rotated.30 <- rotate_points(pos.original, angle_degrees = 30)
pos.rotated.60 <- rotate_points(pos.original, angle_degrees = 60)
pos.rotated.90 <- rotate_points(pos.original, angle_degrees = 90)
```

The original gene expression pattern for Gene 31:

```r
gene_expression <- counts.st[31,]
scaled_gene_expression <- (gene_expression - min(gene_expression)) /
  (max(gene_expression) - min(gene_expression))
dat.st.original <- data.frame(x = pos.original[,1],
                              y = pos.original[,2],
                              gene_expression = scaled_gene_expression
                              )
ggplot(dat.st.original, aes(x, y, color = gene_expression)) +
  geom_point() +
  theme_minimal() +
  scale_color_continuous(low = "cornsilk", high = "darkred") +
  theme(legend.title = element_text(size = 11, face = "bold"),
        axis.text = element_blank(),
        panel.grid.major = element_blank(),
        panel.grid.minor = element_blank()) +
  labs(color = "Scaled gene expression", title = "Original pattern") +
  guides(color = guide_legend(override.aes = list(size = 4)))
```

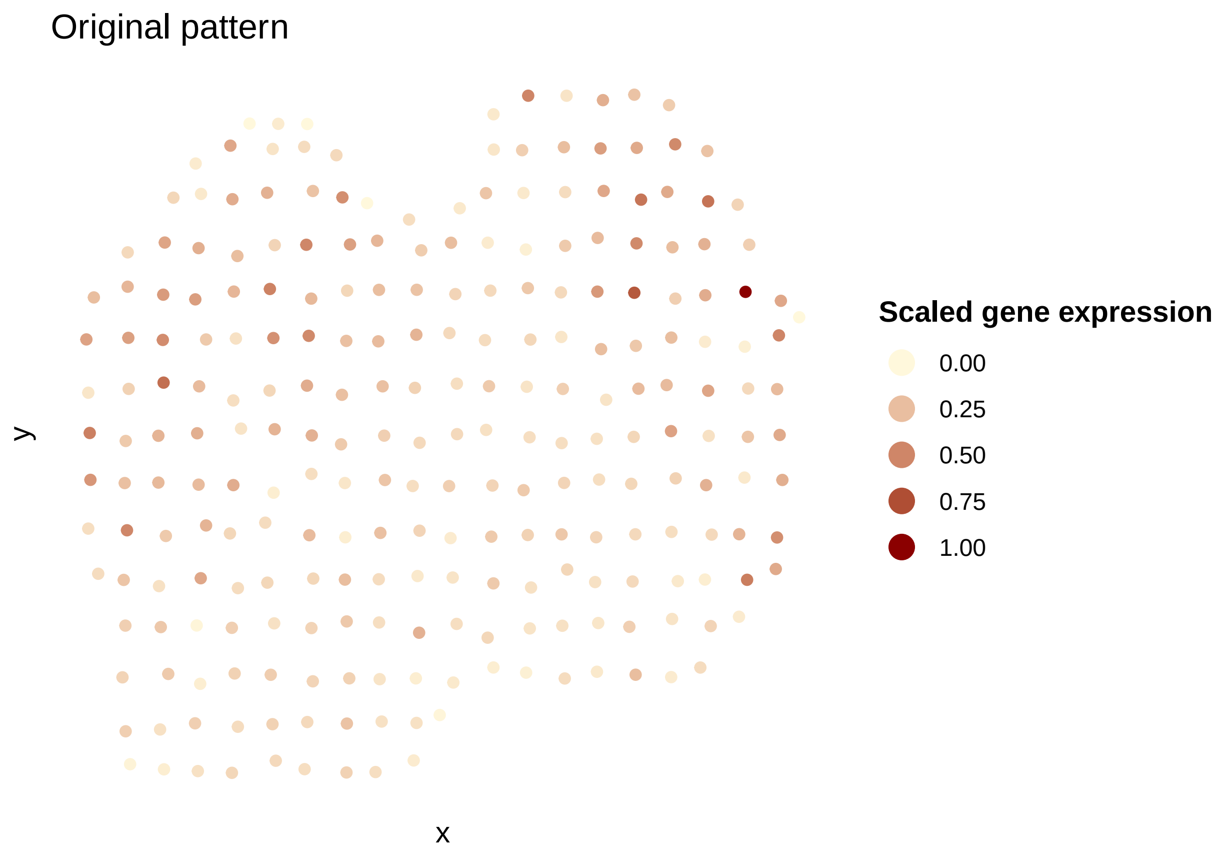

The rotated gene expression pattern for Gene 31:

```
par(mfrow = c(1, 3), pty = "s")
dat.st.rotated.30 <- data.frame(x = pos.rotated.30[,1],
                                y = pos.rotated.30[,2],
                                gene_expression = scaled_gene_expression
                                )
ggplot(dat.st.rotated.30, aes(x, y, color = gene_expression)) +
  geom_point() +
  theme_minimal() +
  scale_color_continuous(low = "cornsilk", high = "darkred") +
  theme(legend.title = element_text(size = 11, face = "bold"),
        axis.text = element_blank(),
        panel.grid.major = element_blank(),
        panel.grid.minor = element_blank()) +
  labs(color = "Scaled gene expression", title = "30-degree rotated pattern") +
  guides(color = guide_legend(override.aes = list(size = 4)))
```

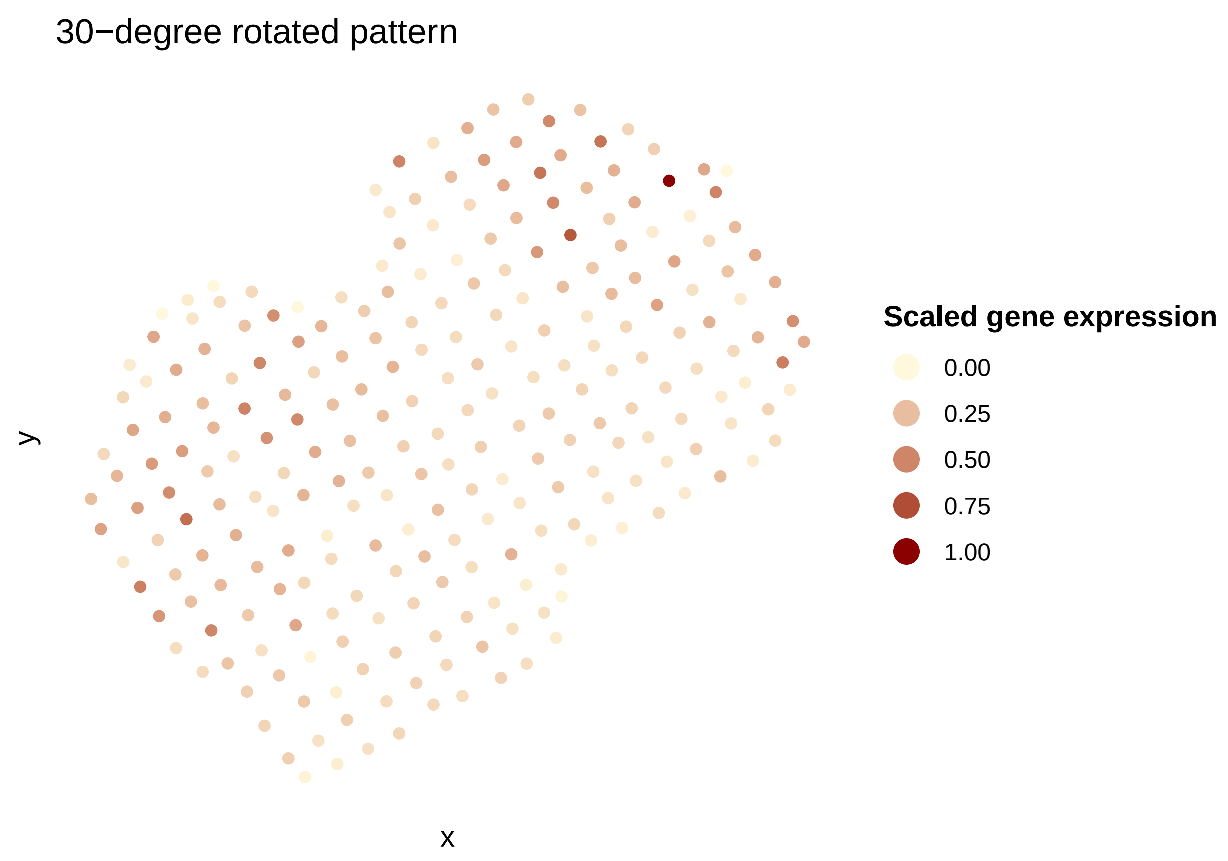


```
dat.st.rotated.60 <- data.frame(x = pos.rotated.60[,1],
                                y = pos.rotated.60[,2],
                                gene_expression = scaled_gene_expression
                                )
ggplot(dat.st.rotated.60, aes(x, y, color = gene_expression)) +
  geom_point() +
  theme_minimal() +
  scale_color_continuous(low = "cornsilk", high = "darkred") +
  theme(legend.title = element_text(size = 11, face = "bold"),
        axis.text = element_blank(),
        panel.grid.major = element_blank(),
        panel.grid.minor = element_blank()) +
  labs(color = "Scaled gene expression", title = "60-degree rotated pattern") +
  guides(color = guide_legend(override.aes = list(size = 4)))
```

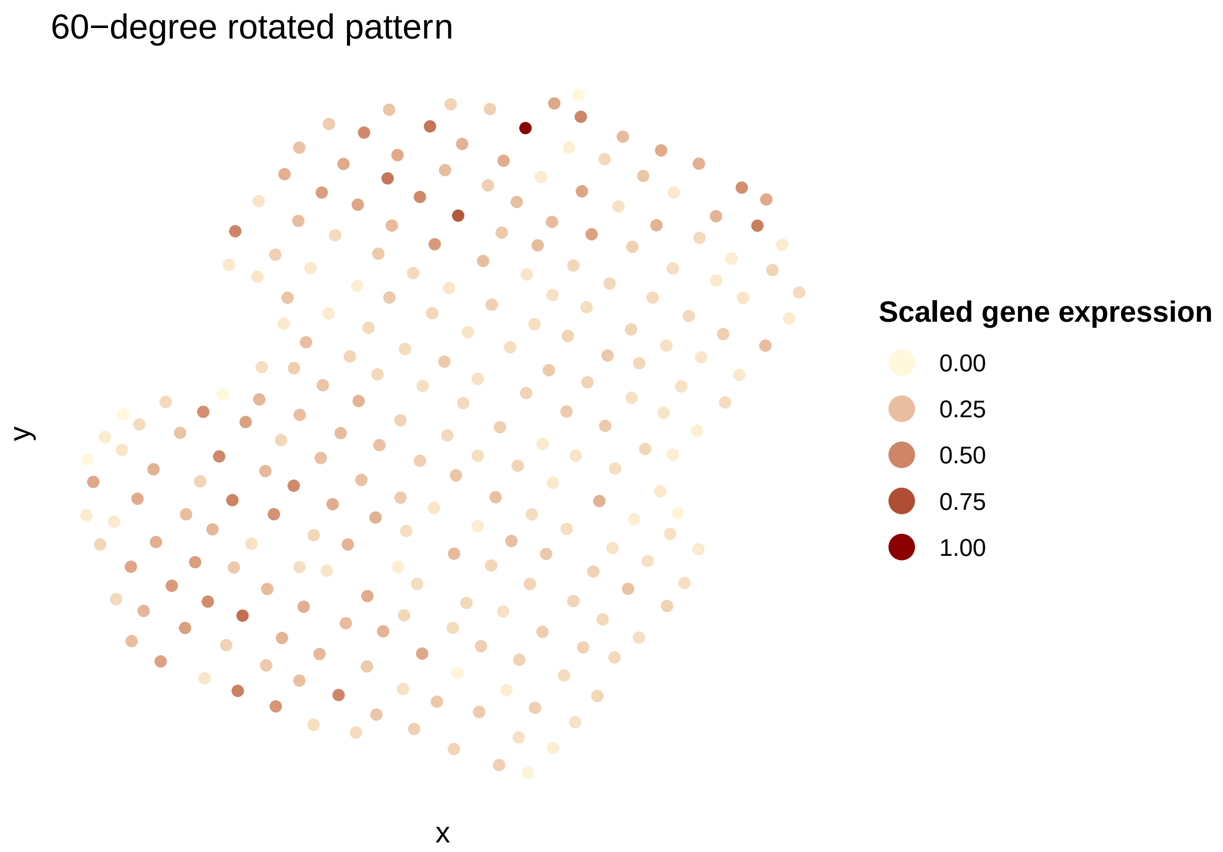


```r
dat.st.rotated.90 <- data.frame(x = pos.rotated.90[,1],
                                y = pos.rotated.90[,2],
                                gene_expression = scaled_gene_expression
                                )
ggplot(dat.st.rotated.90, aes(x, y, color = gene_expression)) +
  geom_point() +
  theme_minimal() +
  scale_color_continuous(low = "cornsilk", high = "darkred") +
  theme(legend.title = element_text(size = 11, face = "bold"),
        axis.text = element_blank(),
        panel.grid.major = element_blank(),
        panel.grid.minor = element_blank()) +
  labs(color = "Scaled gene expression", title = "90-degree rotated pattern") +
  guides(color = guide_legend(override.aes = list(size = 4)))
```

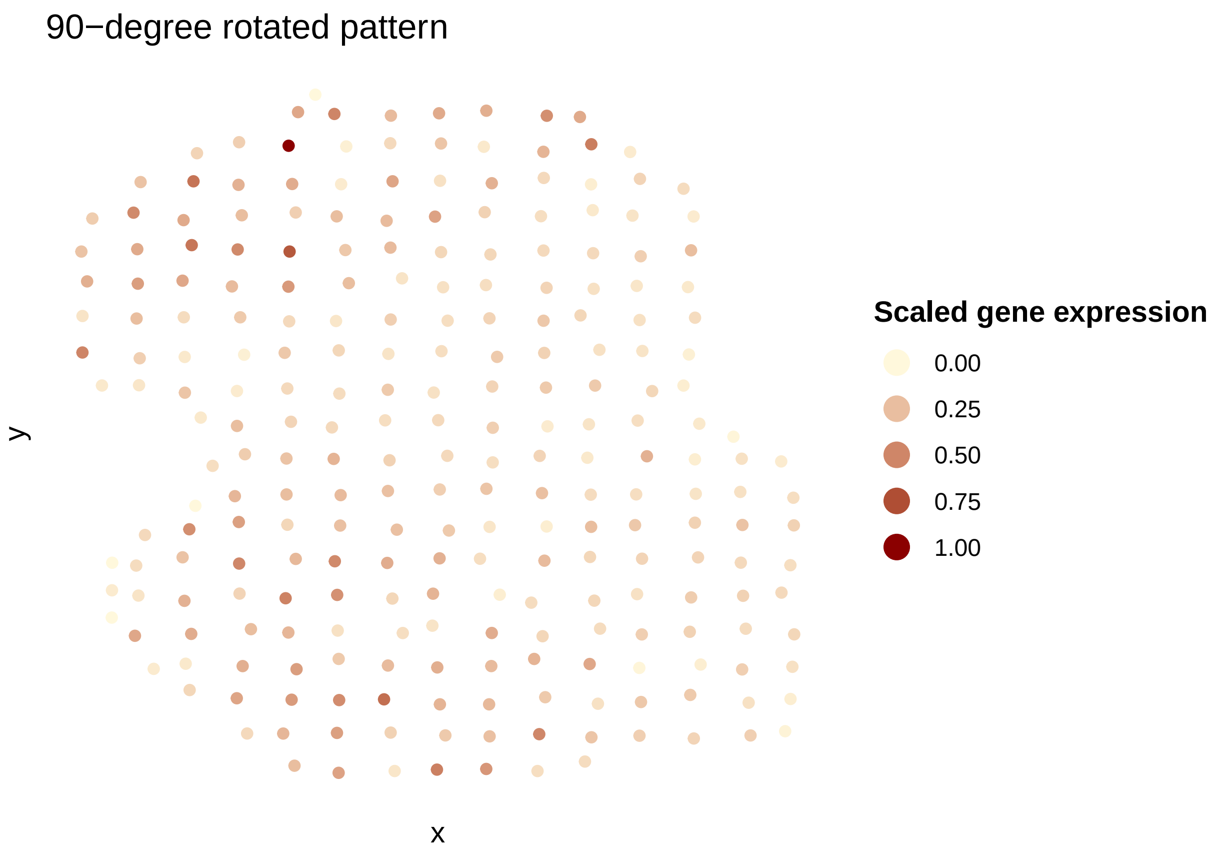

C-SIDE

Next, we analyzed the data with C-SIDE [13] using the data before and after rotation. In theory, the results should be exactly the same given that the relative relationship between gene expressions and their spatial locations remain unchanged. As outlined in the C-SIDE tutorial, the “create.RCTD” and “run.RCTD” functions must be executed before performing C-SIDE. Therefore, we applied RCTD[1] to both the original and rotated patterns to create the RCTD objects for all the patterns.

```
Cell_Types <- as.factor(Cell_Types)
names(Cell_Types) <- paste0("Cell", 1:length(Cell_Types))
# Create the Reference object
RCTD_reference <- Reference(counts = counts.sc,
                            cell_types = Cell_Types, min_UMI = 1)
# Create the SpatialRNA object for the original pattern
RCTD_puck.original <- SpatialRNA(coords = as.data.frame(pos.original),
                                 counts = counts)
# Create the RCTD object for the original pattern
myRCTD.original <- create.RCTD(RCTD_puck.original, RCTD_reference, max_cores = 1)
# Run RCTD for the original pattern
myRCTD.original <- run.RCTD(myRCTD.original, doublet_mode = "full")
# Import the true cell type composition
myRCTD.original <- import_weights(myRCTD.original, weights = prop)
```

```r
# Create the SpatialRNA object for the 30-degree rotated pattern
RCTD_puck.rotated.30 <- SpatialRNA(coords = as.data.frame(pos.rotated.30),
                                   counts = counts)
# Create the RCTD object for the 30-degree rotated pattern
myRCTD.rotated.30 <- create.RCTD(RCTD_puck.rotated.30, RCTD_reference, max_cores = 1)
# Run RCTD for the 30-degree rotated pattern
myRCTD.rotated.30 <- run.RCTD(myRCTD.rotated.30, doublet_mode = "full")
# Import the 30-degree true cell type composition
myRCTD.rotated.30 <- import_weights(myRCTD.rotated.30, weights = prop)

# Create the SpatialRNA object for the 60-degree rotated pattern
RCTD_puck.rotated.60 <- SpatialRNA(coords = as.data.frame(pos.rotated.60),
                                   counts = counts)
# Create the RCTD object for the 60-degree rotated pattern
myRCTD.rotated.60 <- create.RCTD(RCTD_puck.rotated.60, RCTD_reference, max_cores = 1)
# Run RCTD for the 60-degree rotated pattern
myRCTD.rotated.60 <- run.RCTD(myRCTD.rotated.60, doublet_mode = "full")
# Import the 60-degree true cell type composition
myRCTD.rotated.60 <- import_weights(myRCTD.rotated.60, weights = prop)

# Create the SpatialRNA object for the 90-degree rotated pattern
RCTD_puck.rotated.90 <- SpatialRNA(coords = as.data.frame(pos.rotated.90),
                                   counts = counts)
# Create the RCTD object for the 30-degree rotated pattern
myRCTD.rotated.90 <- create.RCTD(RCTD_puck.rotated.90, RCTD_reference, max_cores = 1)
# Run RCTD for the 30-degree rotated pattern
myRCTD.rotated.90 <- run.RCTD(myRCTD.rotated.90, doublet_mode = "full")
# Import the 30-degree true cell type composition
myRCTD.rotated.90 <- import_weights(myRCTD.rotated.90, weights = prop)
```

We can verify that the estimated cell type compositions by RCTD remain invariant to rotation by the following step for validation.

```r
# Check whether the estimated cell type compositions matrices from rotated patterns
# are the same as the one from the original pattern.
all(myRCTD.original@results$weights == myRCTD.rotated.30@results$weights)
```

```
## [1] TRUE
```

```r
all(myRCTD.original@results$weights == myRCTD.rotated.60@results$weights)
```

```
## [1] TRUE
```

```r
all(myRCTD.original@results$weights == myRCTD.rotated.90@results$weights)
```

```
## [1] TRUE
```

Run CSIDE using `run.CSIDE.nonparam` for both original and rotated patterns.

```r
# Run CSIDE for the original pattern
CSIDE.results.original <- run.CSIDE.nonparam(myRCTD.original, df = 6,
                                      cell_types = paste0('CT',1:3),
                                      gene_threshold = .001,
                                      cell_type_threshold = 10,
                                      fdr = 0.01, doublet_mode = FALSE)
# Run CSIDE for the rotated pattern
CSIDE.results.rotated.30 <- run.CSIDE.nonparam(myRCTD.rotated.30, df = 6,
                                     cell_types = paste0('CT',1:3),
                                     gene_threshold = .001,
                                     cell_type_threshold = 10,
                                     fdr = 0.01, doublet_mode = FALSE)
# Run CSIDE for the rotated pattern
CSIDE.results.rotated.60 <- run.CSIDE.nonparam(myRCTD.rotated.60, df = 6,
                                     cell_types = paste0('CT',1:3),
                                     gene_threshold = .001,
                                     cell_type_threshold = 10,
                                     fdr = 0.01, doublet_mode = FALSE)
# Run CSIDE for the rotated pattern
CSIDE.results.rotated.90 <- run.CSIDE.nonparam(myRCTD.rotated.90, df = 6,
                                     cell_types = paste0('CT',1:3),
                                     gene_threshold = .001,
                                     cell_type_threshold = 10,
                                     fdr = 0.01, doublet_mode = FALSE)
```

Then, we checked the significant gene list for cell type 1 (CT1).

```r
print(CSIDE.results.original@de_results$sig_gene_list$CT1)
```

```
##          Z_score   log_fc        se paramindex_best conv        p_val
## Gene36 4.674277 1.852129 0.3962386               3 TRUE 1.474955e-05
## Gene34 3.895407 2.637814 0.6771600               3 TRUE 4.901690e-04
## Gene37 0.000000 0.000000 0.0000000               0 TRUE 6.635030e-04
```

```r
print(CSIDE.results.rotated.30@de_results$sig_gene_list$CT1)
```

```
##          Z_score    log_fc        se paramindex_best conv        p_val
## Gene36 4.604733 -2.445881 0.5311668               2 TRUE 0.0000206498
## Gene39 3.874766 -2.798021 0.7221137               2 TRUE 0.0005336364
## Gene34 0.000000  0.000000 0.0000000               0 TRUE 0.0005563409
```

```r
print(CSIDE.results.rotated.60@de_results$sig_gene_list$CT1)
```

```
##          Z_score    log_fc        se paramindex_best conv        p_val
## Gene36 4.785549 -2.400817 0.5016806               2 TRUE 8.526048e-06
## Gene39 3.975769 -2.391142 0.6014288               2 TRUE 3.507611e-04
## Gene37 3.712272 -1.588727 0.4279663               2 TRUE 1.027037e-03
## Gene34 0.000000  0.000000 0.0000000               0 TRUE 1.170519e-03
```

```r
print(CSIDE.results.rotated.90@de_results$sig_gene_list$CT1)
```

```
##         Z_score    log_fc        se paramindex_best conv        p_val
## Gene36 4.674277 -1.852129 0.3962386               2 TRUE 1.474955e-05
## Gene34 3.895407 -2.637814 0.6771600               2 TRUE 4.901690e-04
## Gene37 0.000000  0.000000 0.0000000               0 TRUE 6.635031e-04
```

Gene 37 is identified as a CT1-specific ctSVG under the original, 60-degree, and 90-degree rotated patterns but is no longer significant after a 30-degree rotation, indicating a false negative by C-SIDE for this gene at that angle. Similarly, Gene 39 is identified as a CT1-specific ctSVG after 30-degree and 60-degree rotations but is not significant under the original or 90-degree rotated patterns. These inconsistencies highlight that the testing results of C-SIDE are not invariant to spatial rotations.

spVC

spVC [14] uses the bivariate penalized spline over triangulation (BPST) method to approximate cell type-specific spatial effects, which requires pre-selected boundary points. We pre-selected the boundary points for this sample pattern and saved as `Sample_stBoundary.rda` (available at https://drive.google.com/drive/folders/1KSxeInbwFswuJdTBxCjMZKc6voz5UWqZ?usp=drive_link). Next we loaded the file and used TriMesh to create trigangulations for the original and rotated patterns.

```r
## For the original pattern
load(file = 'Sample_stBoundary.rda') # Load the pre-selected boundary points
# TriMesh is used to create triangulation
Tr.cell.original <- TriMesh(mouse.cerebellum.boundary.original, n = 2)
V.original <- as.matrix(Tr.cell.original$V)
Tr.original <- as.matrix(Tr.cell.original$Tr)
# The boundary points of 30-degree rotated pattern
mouse.cerebellum.boundary.rotated.30 <- rotate_points(mouse.cerebellum.boundary.original,
                                                      angle_degrees = 30)
Tr.cell.rotated.30 <- TriMesh(mouse.cerebellum.boundary.rotated.30, n = 2)
V.rotated.30 <- as.matrix(Tr.cell.rotated.30$V)
Tr.rotated.30 <- as.matrix(Tr.cell.rotated.30$Tr)

# The boundary points of 60-degree rotated pattern
mouse.cerebellum.boundary.rotated.60 <- rotate_points(mouse.cerebellum.boundary.original,
                                                      angle_degrees = 60)
Tr.cell.rotated.60 <- TriMesh(mouse.cerebellum.boundary.rotated.60, n = 2)
V.rotated.60 <- as.matrix(Tr.cell.rotated.60$V)
Tr.rotated.60 <- as.matrix(Tr.cell.rotated.60$Tr)

# The boundary points of 90-degree rotated pattern
mouse.cerebellum.boundary.rotated.90 <- rotate_points(mouse.cerebellum.boundary.original,
                                                      angle_degrees = 90)
Tr.cell.rotated.90 <- TriMesh(mouse.cerebellum.boundary.rotated.90, n = 2)
V.rotated.90 <- as.matrix(Tr.cell.rotated.90$V)
Tr.rotated.90 <- as.matrix(Tr.cell.rotated.90$Tr)
```

Then, spVC was run for both data before and after rotation.

```r
# Fit the spVC model for the original pattern
spVC.results.original <- test.spVC(Y = counts, X = prop, S = pos.original,
                                   V = V.original, Tr = Tr.original,
                                   para.cores = 1, filter.min.nonzero = 5)
```

```
## spVC model will use  94.42231 % of the original data.
## Conducting tests for 50  genes.
## Model 2: Conducting tests for 1 genes.
```

```r
# Fit the spVC model for the 30-degree rotated pattern
spVC.results.rotated.30 <- test.spVC(Y = counts, X = prop, S = pos.rotated.30,
                                     V = V.rotated.30, Tr = Tr.rotated.30,
                                     para.cores = 1, filter.min.nonzero = 5)
```

```
## spVC model will use  94.42231 % of the original data.
## Conducting tests for 50  genes.
## Model 2: Conducting tests for 2 genes.
```

```r
# Fit the spVC model for the 60-degree rotated pattern
spVC.results.rotated.60 <- test.spVC(Y = counts, X = prop, S = pos.rotated.60,
                                     V = V.rotated.60, Tr = Tr.rotated.60,
                                     para.cores = 1, filter.min.nonzero = 5)
```

```
## spVC model will use  94.42231 % of the original data.
## Conducting tests for 50  genes.
## Model 2: Conducting tests for 1 genes.
```

```r
# Fit the spVC model for the 90-degree rotated pattern
spVC.results.rotated.90 <- test.spVC(Y = counts, X = prop, S = pos.rotated.90,
                                     V = V.rotated.90, Tr = Tr.rotated.90,
                                     para.cores = 1, filter.min.nonzero = 5)
```

```
## spVC model will use  94.42231 % of the original data.
## Conducting tests for 50  genes.
## Model 2: Conducting tests for 2 genes.
```

We checked the testing results for one of the CT1-specific ctSVG. Here, we selected Gene 37 as an example.

```r
# spVC
print(spVC.results.original$results.constant$Gene37$p.value)
```

```
##       beta_0      beta_X1      beta_X2      beta_X3      gamma_0
## 5.392343e-30 2.094877e-04          NaN 2.269201e-01 1.337169e-01
```

```r
print(spVC.results.rotated.30$results.constant$Gene37$p.value)
```

```
##       beta_0      beta_X1      beta_X2      beta_X3      gamma_0
## 2.386003e-39 1.036450e-02 2.790764e-01          NaN 6.185813e-02
```

```r
print(spVC.results.rotated.60$results.constant$Gene37$p.value)
```

```
##       beta_0      beta_X1      beta_X2      beta_X3      gamma_0
## 1.275762e-40 1.109691e-02 2.220875e-01          NaN 4.756965e-01
```

```r
print(spVC.results.rotated.90$results.constant$Gene37$p.value)
```

```
##       beta_0      beta_X1      beta_X2      beta_X3      gamma_0
## 1.264229e-39 6.387597e-03 2.359197e-01          NaN 1.782484e-10
```

Gene 37 passed the stage 1 test under the original and 30-degree rotated patterns, since the p-values for testing the cell-type-1-associated spatially constant effect `beta_X1` and spatially varying effect `gamma_0` were significant at the 0.05 level. On the other hand, it did not pass the stage 1 test after 60-degree and 90-degree rotation, since none of p-values for testing the cell-type-associated spatially constant effects `beta_X1`, `beta_X2`, `beta_X3` was significant at the 0.05 level, even though the residual spatial effect `gamma_0` was significant. This shows that the testing results of spVC are not invariant to spatial rotation.

CTSV

Finally, we performed CTSV[12] on the data before and after rotation.

```r
spe.original <- SpatialExperiment(assay = counts[,which(colSums(counts) != 0)],
                                  colData = pos.original,
                                  spatialCoordsNames = c('x', 'y'))
spe.rotated.30 <- SpatialExperiment(assay = counts[,which(colSums(counts) != 0)],
                                    colData = pos.rotated.30,
                                    spatialCoordsNames = c('x', 'y'))
spe.rotated.60 <- SpatialExperiment(assay = counts[,which(colSums(counts) != 0)],
                                    colData = pos.rotated.60,
                                    spatialCoordsNames = c('x', 'y'))
spe.rotated.90 <- SpatialExperiment(assay = counts[,which(colSums(counts) != 0)],
                                    colData = pos.rotated.90,
                                    spatialCoordsNames = c('x', 'y'))

CTSV.results.original <- CTSV(spe.original, W = prop, num_core = 1)
CTSV.results.rotated.30 <- CTSV(spe.rotated.30, W = prop, num_core = 1)
CTSV.results.rotated.60 <- CTSV(spe.rotated.60, W = prop, num_core = 1)
CTSV.results.rotated.90 <- CTSV(spe.rotated.90, W = prop, num_core = 1)
```

We checked the significant gene list for cell type 1 (CT1).

```r
print(svGene(CTSV.results.original$qval, 0.05)$SVGene[[1]])
```

```
## [1] "Gene13" "Gene32" "Gene34" "Gene40"
```

```
print(svGene(CTSV.results.rotated.30$qval, 0.05)$SVGene[[1]])
```

```
## [1] "Gene32" "Gene34" "Gene37" "Gene38" "Gene39" "Gene40"
```

```
print(svGene(CTSV.results.rotated.60$qval, 0.05)$SVGene[[1]])
```

```
##  [1] "Gene17" "Gene30" "Gene32" "Gene33" "Gene34" "Gene35" "Gene36" "Gene37"
##  [9] "Gene38" "Gene39" "Gene40"
```

```
print(svGene(CTSV.results.rotated.90$qval, 0.05)$SVGene[[1]])
```

```
## [1] "Gene13" "Gene32" "Gene34" "Gene40"
```

The gene lists for CT1-specific ctSVGs differ across various spatial rotation patterns, indicating that CTSV is not invariant to spatial rotation.

Summary

In summary, we simulated 10 CT1-specific ctSVGs and applied the CTSV, C-SIDE and spVC to the data before and after 30-degree, 60-degree as well as 90-degree spatial rotation. We observed inconsistencies in the testing results before and after rotation for CTSV, C-SIDE and spVC, even though the results should have remained unchanged. Furthermore, as the rotation angle varied, different testing outcomes were observed. These inconsistent results highlight that these methods are unreliable for ctSVG detection and should be avoided in ctSVG analysis.

Supplementary References

1. Cable, D. M. *et al.* Robust decomposition of cell type mixtures in spatial transcriptomics. *Nature Miotechnology* **40,** 517–526 (2022).
2. Ma, Y. & Zhou, X. Spatially informed cell-type deconvolution for spatial transcriptomics. *Nature Biotechnology* **40,** 1349–1359 (2022).
3. Miller, B. F., Huang, F., Atta, L., Sahoo, A. & Fan, J. Reference-free cell type deconvolution of multi-cellular pixel-resolution spatially resolved transcriptomics data. *Nature Communications* **13,** 2339 (2022).
4. Searle, S. R., Casella, G. & McCulloch, C. E. *Variance components* (John Wiley & Sons, 2009).
5. Dodge, Y. *The Oxford dictionary of statistical terms* (Oxford University Press, USA, 2003).
6. Bartlett, M. S. Properties of sufficiency and statistical tests. *Proceedings of the Royal Society of London. Series A-Mathematical and Physical Sciences* **160,** 268–282 (1937).
7. Gilmour, A. R., Thompson, R. & Cullis, B. R. Average information REML: an efficient algorithm for variance parameter estimation in linear mixed models. *Biometrics,* 1440–1450 (1995).
8. Perdry, H., Dandine-Roulland, C., Bandyopadhyay, D. & Kettner, L. gaston: Genetic data handling (QC, GRM, LD, PCA) & linear mixed models. *R package version* **1** (2018).
9. Zhang, D. & Lin, X. Hypothesis testing in semiparametric additive mixed models. *Biostatistics* **4,** 57–74 (2003).
10. Liu, D., Lin, X. & Ghosh, D. Semiparametric regression of multidimensional genetic pathway data: least-squares kernel machines and linear mixed models. *Biometrics* **63,** 1079–1088 (2007).
11. Satterthwaite, F. E. An approximate distribution of estimates of variance components. *Biometrics Bulletin* **2,** 110–114 (1946).
12. Yu, J. & Luo, X. Identification of cell-type-specific spatially variable genes accounting for excess zeros. *Bioinformatics* **38,** 4135–4144 (2022).
13. Cable, D. M. *et al.* Cell type-specific inference of differential expression in spatial transcriptomics. *Nature Methods* **19,** 1076–1087 (2022).
14. Yu, S. & Li, W. V. spVC for the detection and interpretation of spatial gene expression variation. *Genome Biology* **25,** 103 (2024).
15. Shang, L. & Zhou, X. Spatially aware dimension reduction for spatial transcriptomics. *Nature Communications* **13,** 7203 (2022).
16. Sheather, S. J. & Jones, M. C. A reliable data-based bandwidth selection method for kernel density estimation. *Journal of the Royal Statistical Society: Series B (Methodological)* **53,** 683–690 (1991).
17. Silverman, B. W. *Density estimation for statistics and data analysis* (Routledge, 2018).

18. Andersson, A. *et al.* Spatial deconvolution of HER2-positive breast cancer delineates tumor-associated cell type interactions. *Nature Communications* **12,** 6012 (2021).

19. Andersson, A. *et al. Single-cell and spatial transcriptomics enables probabilistic inference of cell type topography* 2020.

20. Ståhl, P. L. *et al.* Visualization and analysis of gene expression in tissue sections by spatial transcriptomics. *Science* **353,** 78–82 (2016).

21. Raudvere, U. *et al.* g: Profiler: a web server for functional enrichment analysis and conversions of gene lists (2019 update). *Nucleic Acids Research* **47,** W191–W198 (2019).

22. Sun, S., Zhu, J. & Zhou, X. Statistical analysis of spatial expression patterns for spatially resolved transcriptomic studies. *Nature Methods* **17,** 193–200 (2020).

23. Zhu, J., Sun, S. & Zhou, X. SPARK-X: non-parametric modeling enables scalable and robust detection of spatial expression patterns for large spatial transcriptomic studies. *Genome Biology* **22,** 184 (2021).

24. Zhu, J., Shang, L. & Zhou, X. SRTsim: spatial pattern preserving simulations for spatially resolved transcriptomics. *Genome Biology* **24**, 39 (2023).
